# Two rare flavonoid glycosides from *Litsea glutinosa* (Lour.) C. B. Rob.: experimental and computational approaches endorse antidiabetic potentiality

**DOI:** 10.1186/s12906-024-04337-0

**Published:** 2024-02-01

**Authors:** Israt Jahan Bulbul, Md. Jamal Hossain, Mohammad Rashedul Haque, Muhammad Abdullah Al-Mansur, Choudhury M. Hasan, Abdullah Al Hasan, Mohammad A. Rashid

**Affiliations:** 1 Department of Pharmacy, Southeast University, 252, Tejgaon Industrial Area, Dhaka 1208 Bangladesh; 2 Department of Pharmacy, School of Pharmaceutical Sciences, State University of Bangladesh, South Purbachal, Kanchan, Dhaka, 1461 Bangladesh; 3 Phytochemical Research Laboratory, Department of Pharmaceutical Chemistry, Faculty of Pharmacy, University of Dhaka, Dhaka, 1000 Bangladesh; 4 Bangladesh Council of Scientific and Industrial Research (BCSIR), Dr. Qudrat-I-Khuda Road, Dhanmondi, Dhaka, 1205 Bangladesh

**Keywords:** *Litsea glutinosa*, Flavonoid glycosides, Molecular docking, Antidiabetic effect, ADMET analyses, PASS Prediction

## Abstract

**Background:**

*Litsea glutinosa* (Lour.) C. B. Rob. belongs to the *Litsea* genus and is categorized under the family of *Lauraceae*. The study aimed to investigate the phytoconstituents and pharmacological properties of methanol extract of leaves of *Litsea glutinosa*, focusing on antidiabetic activity via in vivo and in silico techniques.

**Methods:**

Extensive chromatographic and spectroscopic techniques were applied to isolate and characterize the constituents from the *L. glutinosa* plant species. The antidiabetic activity was studied in streptozotocin-induced diabetes mice, and the computational study of the isolated compounds was carried out by utilizing AutoDock Vina programs. In addition, the pharmacokinetic properties in terms of absorption, distribution, metabolism and excretion (ADME) and toxicological profiles of the isolated compounds were examined via in silico techniques.

**Results:**

In the present study, two flavonoid glycosides 4΄-*O*-methyl (2 ˝,4 ˝-di-*E*-p-coumaroyl) afzelin (1) and quercetin 3-*O*-(2 ˝,4 ˝-di-*E*-p-coumaroyl)-α-L-rhamnopyranoside (2) were isolated from the leaves of *L. glutinosa* and characterized by ^1^H and ^13^C NMR, COSY, HSQC, HMBC, and mass spectral data. Although compounds 1 and 2 have been reported twice from *Machilis litseifolia* and *Lindera akoensis*, and *Machilis litseifolia* and *Mammea longifolia*, respectively, this is the first report of this isolation from a *Litsea* species. Administering the methanolic extract of *L. glutinosa* at doses of 300 and 500 mg/kg/day to mice with diabetes induced by streptozotocin led to a significant decrease in fasting blood glucose levels (*p* < 0.05) starting from the 7th day of treatment. Besides, the computational study and PASS analysis endorsed the current in vivo findings that the both isolated compounds exerted higher binding affinities to human pancreatic α-amylase and aldose reductase than the conventional drugs. The in silico ADMET analysis revealed that the both isolated compounds have a favorable pharmacokinetic and safety profile suitable for human consumption.

**Conclusion:**

According to the current outcomes obtained from in vivo and in silico techniques, the leaf extract of *L. glutinosa* could be a natural remedy for treating diabetes, and the isolated phytoconstituents could be applied against various illnesses, mainly hyperglycemia. However, more investigations are required for extensive phytochemical isolation and pharmacological activities of these phytoconstituents against broader targets with exact mechanisms of action.

**Supplementary Information:**

The online version contains supplementary material available at 10.1186/s12906-024-04337-0.

## Background

Diabetes Mellitus (DM) is heterogeneous in respect of its pathogenesis, clinical features and affecting or contributing genetic factors, characterized by persistent hyperglycaemia and multiple organ level disorders i.e., the progression of eye, kidney, and nerve complications due to the increased blood sugar level for longer period [1]. Diabetes mellitus has emerged as a serious health issue worldwide that causes significant morbidity and mortality. The International Diabetes Federation reports that approximately 537 million individuals between the ages of 20 and 79 years have diabetes, which accounts for 10.5% of the world's population in this age group. The estimated figures indicate that there will be a rise in the number of individuals affected by diabetes, with projections showing a potential increase to 643 million people (equivalent to 11.3% of the population) by 2030 and a further rise to 783 million individuals (around 12.2% of the population) by 2045 [2]. Present therapies often fail to sustain glucose level as well as to reduce diabetes-related complications in the long-term. Treatment of diabetes using herbs and plants is becoming very popular worldwide due to minimum or no side effects, absent or low toxicity, comprehensive biodegradability, easy availability as compared with the insufficient supply, unaffordable treatment cost and severe side effects of synthetic drugs [3]. Bioactive chemicals are abundant in nature, and from 1981 to 2014, substances from nature served as the basis or influence for 51% of approved medications [4]. Several natural products that possess the ability to inhibit α-glucosidase have been discovered. Among these, two natural products are acarbose and voglibose, which are two useful inhibitors of α-glucosidase with therapeutic applications [5].

*Litsea glutinosa* (Lour.) C. B. Rob. is a species that is classified within the Lauraceae family and is a part of the *Litsea* genus. In the Lauraceae family, *Litsea* is a large genus which is the second larger one than the *Octea*. The species of *Litsea* genus form a vital element of tropical forests. Among more than 300 species, most of them are found in tropical Asia and a few species are habitat in Australia, the islands of the Pacific and in Central and North America [6, 7]. In Bangladesh, there are 11 species of *Litsea* as recorded by the Ministry of Environment, Forest and Climate Change [8, 9]. *L. glutinosa* is indigenous to all over Asia, containing China, Bhutan, India, Nepal, Myanmar, Vietnam, Thailand, the Philippines, Bangladesh, Hong Kong, Indonesia, Malaysia, Laos, Cambodia, Papua New Guinea, the Solomon Islands. It is also native to Northern Territory, Western Australia, Cape York Peninsula, southern end and northeast Queensland [10].

In ethnomedicine, roots, bark, leaves and seeds of *L. glutinosa* are considered as therapeutically important. Its leaves and bark are used for diarrhea and dysentery, and its root paste is applied as poultice for bruises and sprains in India [11]. Chopped and soaked leaves are used as plaster in the Northern Philippines. *L. glutinosa* is a tree with low density wood, so it is used as fuel wood [12]. In China, the seed oil is used for making soap [13]. In Mayotte (Indian Ocean), *L. glutinosa* is used as a fodder tree to feed 93% of the cattle there [14]. In addition, evidence found that this plant had various traditional applications, including treating conditions like diarrhea, dysentery, abdominal discomfort, digestive issues, gastroenteritis, swelling, injuries, common colds, arthritis, asthma, diabetes, alleviating pain, and enhancing sexual potency [11–14].

A crude methanol extract of *L. glutinosa* leaves was reported to have thrombolytic, anti-inflammatory, analgesic and antipyretic activities [15]. The methanolic bark extract of the plant species showed the ability to fight against both types of bacteria, gram-negative and gram-positive [16]. Besides, the methanol extract of bark was also reported to have potent hepatoprotective action against liver damage in rats induced by paracetamol and CCl_4_ [17]. According to Palanuvej et al. [18], the leaves of this plant species produce mucilage, which has been found to possess anti-diabetic and antioxidant properties. On the other hand, traditional and tribal healers use the oil extracted from the berries of this plant as a remedy for rheumatism [18]. This plant is utilized in ancient Chinese medicinal practices to address diabetes and inflammation. Numerous research studies have provided evidence of its effectiveness in combating diabetes, reducing inflammation, and displaying antibacterial properties [19, 20]. Sun et al., [21] examined the impact of an aminoethylstilbene isoquinoline alkaloid called litsine C, derived from the root bark of *L. glutinosa*, and tested on glucose consumption in HepG2 cells. Various concentrations of litsine C (ranging from 1 to 20 μM) were utilized, and it was observed that the compound litsine C notably enhanced glucose uptake. Additionally, litsine A, which was obtained from the root barks of *L. glutinosa*, demonstrated an elevation in glucose absorption when tested on C2C12 myoblasts in the glucose-uptake assay at a concentration of 10 μM [22]. In an another study conducted by Zhang et al., [23] it was found that administering ob/ob mice an alkaloid-rich extract derived from the barks of *L. glutinosa* orally at doses of 50, 100, and 200 mg/kg for a duration of 4 weeks resulted in promising effects in reducing high blood sugar levels and high lipid levels. The study suggested that the bark extract has the potential to be used as an effective treatment for type 2 diabetes.

Several alkaloids for example isoboldine, actinodaphnine, liriodenine, laureliptine, nmethyl actinodaphnine, laurotetanine, boldine, n-methyllaurotetanine, laurolitsine, litsine, litseferine and glutinosine A were reported from the plant species [11, 20, 22]. Furthermore, this plant has already been reported to contain certain compounds like flavonoids, such as 2',5,7-trihydroxy-6-methoxy flavone 2'-O-beta-D glucopyranoside [24], sesquiterpenes, such as β-caryophyllene, caryophyllene oxide, and monoterpenes like (E)-β-Ocimene and (Z)-β-Ocimene [17]. Likewise, multiple research studies have reported different types of compounds derived from the plant species, and there are numerous fascinating reports about the blood sugar-lowering or anti-diabetic effects of the plant's roots, bark, or derived components.

While there is compelling evidence suggesting that the root extract and its components, as well as the bark extract and its constituents from the plant species, hold promise as potential treatments for diabetes [21–23], there is currently limited information available regarding the antidiabetic properties of the leaf extract of the plant. Therefore, there is a need for extensive evidence concerning the phyto-pharmacological studies related to the plant leaves, including a focus on in vivo antidiabetic properties of the leaf extract of the plant. At this stage, this study aimed to conduct phytochemical, pharmacological, and molecular docking investigations to find and confirm new bioactive lead compounds from the leaves of *L. glutinosa*, highlighting antidiabetic activity.

## Materials and methods

### Collection of plant material

*L. glutinosa* leaves were gathered from Chittagong hilly area of Bangladesh, in accordance with the necessary authorization obtained from the local authority. A taxonomist, Khandakar Kamrul Islam, senior scientific officer of Bangladesh National Herbarium identified and recognized the plant sample, which was then recorded as a voucher specimen with the accession number DACB-37904 in Bangladesh National Herbarium located in Mirpur-1, Dhaka-1216, Bangladesh. The research methods involved with the plant materials was approved by the Research Committee within the Department of Pharmacy at Southeast University, Dhaka, Bangladesh.

### Chemicals and instruments

The Bruker 400 MHz NMR spectrometer was utilized to record both 1D and 2D NMR spectra, with the internal reference standard being tetramethyl silane (TMS). The ppm values have been used to express the chemical shifts (δ), while the coupling constants (J) have been indicated in Hz. An LC–MS/MS System (ESI–MS) from Thermo Scientific in Waltham, MA, USA, was used to conduct electro-spray mass spectroscopy. The method of taking mass spectra involved using HX-110 (JEOL) and/or JMS-700 (JEOL) spectrometers with a direct inlet system, with the exception of cases where it was otherwise specified. Sephadex LH-20 (Qingdao Haiyang Chemical Industry Co., Ltd., Qingdao, China) was used for gel permeation chromatography (GPC). Chromatographic grade n-hexane, chloroform, and methanol were acquired from Chang Tech Enterprise Co., Ltd (Taiwan, China). Kieselgel 60 F254 (Merck) was utilized for preparative TLC. Streptozotocin (STZ) and sucrose were collected from Sigma Chemical Co. (St. Louis, Missouri, USA). In addition, a digital glucometer (yasee, Taiwan) was used for monitoring blood glucose level.

### Extraction and isolation

After collecting, washing with water and drying for some days, all the plant samples were ground to coarse powder by using a high-capacity mill. Then the powdered plant samples (800 gm to 1000 gm) were macerated in methanol (at room temperature) for 7 to 10 days with intermittent shaking. Methanol, having a wide-ranging ability to dissolve numerous phytoconstituents, was utilized in the extraction process due to its universal and potent solvent properties. The extracts of all the plant samples were obtained by filtration through cotton plug and then through filter papers followed by evaporation by using a rotary evaporator to remove excess solvent. Excess of methanol from the extracts was evaporated to get dry mass and retained in the refrigerator for further studies. Solvent–solvent partitioning was done using different solvents with increasing polarity following the procedure by Kupchan which was revised by Van Wagenen et al*.,* [25]. In this method, 5 gm of the crude methanol extract was dissolved in aqueous methanol (10%). Then it was partitioned with petroleum ether, followed by chloroform and ethyl acetate. This process was repetitive with 5 gm methanol extract every time. The fractions were then evaporated to dryness [26, 27]. The yields of different partitionates (Petroleum ether: 3.2 gm, chloroform: 3.7 gm, and ethyl acetate: 1.9 gm) obtained from methanolic extract of *L. glutinosa.* Chloroform soluble fraction of *L. glutinosa* (LGC) was selected for gel permeation chromatography (GPC) followed by the analysis of different column washings by thin layer chromatography (TLC). Then pure compounds were isolated by preparative TLC (PTLC) and characterized by modern spectroscopic methods. GPC is a method of size-exclusion chromatography that utilizes molecular size to isolate bioactive phytoconstituents. The stationary phase comprises of Sephadex LH-20 (porous beads). For suitable swelling dried Sephadex LH-20 was drenched in a mixture of n-hexane- dichloromethane- methanol at 2:5:1 ratio for about 12 h. Then, the slurry of sephadex was added into a glass column 55 cm in height and 1.1 cm in diameter. To ensure a compact packing of the column the solvent system was run several times through the column. Elution was started with a mixture of n-hexane-dichloromethane-methanol at 2:5:1 ratio and the polarity of the solvent system were increased. It is noted that 320 mg of the chloroform fraction of *L. glutinosa* was dissolved in the same solvent mixture as used to pack the column and later applied on top of the packed column by a Pasteur pipette. Depending upon the different solvent systems several sample fractions were eluted and collected in 35 test tubes, each containing about 5 ml of eluted samples. Then each test-tube was analysed by thin layer chromatography (TLC) and then purify by preparative TLC (PTLC) to obtain compound 1 (5.0 mg, EtOAc-Toluene, 50: 50) and compound 2 (3.0 mg, EtOAc-Toluene, 50: 50).

### Animals, acclimatization and ethics

Swiss albino mice of male gender, weighing 30–35 gm and aged 8–10 weeks, were obtained from Jahangirnagar University, Dhaka, Bangladesh. Before the start of the experiment, the mice were housed under standard environmental conditions, maintaining a specific temperature (24 °C ± 1 °C), relative humidity (55% ± 5%), and a 12-h light/dark cycle. Before the experiments began, they were given a week to adjust to their surroundings and were provided with a standard laboratory diet and water ad libitum. All the animals received care and were handled according to the guidelines established by the Swiss Academy of Medical Sciences (SAMS) and the Swiss Academy of Sciences. Besides, the updated guidelines for animal research: reporting in vivo experiments (ARRIVE), were also followed when handling and conducting the animal study [28]. Moreover, the protocols for handling animals and the experimental model were thoroughly reviewed and approved by the Committee on Ethical Compliance in Research of Southeast University, Dhaka, Bangladesh (approval number: SEU/Pharm/CECR/109/2021).

### Experimental design

The study involved two doses (300 and 500 mg/kg b.w.) of the methanol extract of *L. glutinosa*, which were named MELG-1 and MELG-2. The animals were classified into five sets, and each of these groups comprised five mice. This classification was done in a specific way as follows:

- Group I: Normal saline water [0.9% (w/v) NaCl solution BP].
- Group II: Untreated diabetic control group (STZ).
- Group III: STZ + metformin HCl 50 mg/kg b.w.
- Group IV: STZ + 300 mg/kg b.w. MELG.
- Group V: STZ + 500 mg/kg b.w. MELG.

### Sample size calculation

Estimating the appropriate sample size is vital when designing research, even in animal studies. Opting for too few animals might lead to the oversight of significant population differences while selecting an excessive number could result in resource wastage and ethical concerns. As we were unable to ascertain the standard deviation and effect size, we utilized an alternative approach to count the sample size, known as the "resource equation" method [29, 30]. This method establishes an acceptable range for error degrees of freedom (DF) within an analysis of variance (ANOVA) as part of the sample size determination process. In this particular scenario, when dealing with one-way ANOVA, we can estimate the sample size for the error degrees of freedom related to between-subjects variability (also referred to as within-subject degrees of freedom) as follows:

$$n={\text{DF}}/k+1$$

In this context, n represents the number of animals within each group, DF stands for degrees of freedom, and *k* denotes the total number of groups. To determine the minimum and maximum number of animals per group, we considered the acceptable range of the degrees of freedom (DF). We substituted the DF value into our equations using its minimum (10) and maximum (20) values [26]. In this case, we set the value of *k* to 3, representing three test groups, excluding the normal control and disease control groups. As a result, we calculated that the minimum number of animals per group should be approximately 4.33 (calculated from 10/*k* + 1 = 10/3 + 1), while the maximum should be around 7.67 (calculated from 20/*k* + 1 = 20/3 + 1). To ensure consistency, we rounded these values up, resulting in each group containing either five animals for the minimum or seven animals for the maximum to assess the samples' antidiabetic effects. We adjusted the animal count per group up or down as needed to keep the degrees of freedom (DF) within specific boundaries. For example, when there were five animals, we set the DF at 12, and when there were seven animals, we set the DF at 18. This research strictly adhered to the principles of "3R" (Replacement, Reduction, and Refinement) in accordance with Swiss and international guidelines governing the use of animals in experiments [31]. As a result, we chose to use a minimal number of animals (*n* = 5) for this exploratory study, in line with these ethical principles. The entire study was conducted with the utmost commitment to these ethical guidelines.

### Antidiabetic activity

The animals were divided into non-diabetic (Group I) and diabetic (Group II-Group V) groups. Animals of diabetic groups (Group II—Group V) were subjected to diabetic induction with streptozotocin (STZ). Type I diabetes mellitus (TIDM) was induced by administering multiple low-dose of STZ approach, and the procedure was aligned with the protocol described by Furman [32]. Due to the reduced sensitivity of female mice to this toxin-affecting islet cells, we have used male mice in the study [32]. On the first day, all foods were removed from the cage except water, 4 h prior to administration of STZ treatment, for all groups. Then the required amount of STZ was calculated (40 mg/kg/b.w.) for every animal and dissolved in sterile normal saline solution and given 0.5 mL to each animal through intra-peritoneal (i.p.) route of the diabetic groups (Groups II – Group V) by utilizing 1-mL syringes and 25-G needles. The non-diabetic group received only normal saline water at the same volume through i.p. route. Then the animals were returned to their home cages with normal food, water and freshly prepared 10% (w/v) sucrose solution. This procedure was continued for the next four consecutive days, which means that the STZ was administered to each mouse of diabatic groups for five consecutive days. On the following day of the last dose of STZ administration, the 10% (w/v) sucrose solution was replaced with regular water. Afterward, mice were given unrestricted access to a regular diet and water for a week [33]. One week after the last dose of STZ administration, a digital glucometer was used to measure fasted blood glucose levels. Due to mice having a significantly faster metabolic rate than humans, overnight fasting is entirely an extended period, leading to elevated insulin sensitivity [34]. Consequently, the fasting duration was set at 5–6 h while blood glucose levels were being observed [34]. Ethyl chloride was used to produce local anesthesia in the tail and then the tail tip was pricked with the lancing device supplied with the glucometer, followed by squeezing or massaging the tail tip until a bead of blood was developed. Mice with above 8.0 mmol/L fasting blood glucose level (BGL) were chosen for this study [32]. The required amount of standard and selected doses of the extract were calculated rendering the animals’ body weight and dissolved in saline water. It is noted that 0.5 mL solution containing the desired amount of standard and selected doses of the extract was fed each animal for seven consecutive days. Then the fasting BGL was measured at 3rd, 5th and 7th day of the treatment and % inhibition of BGL was calculated by the equation mentioned below:

$$\%\;\mathrm{Inhibition}\;\mathrm{of}\;\mathrm{BGL}=\left({\text{BGL}}_{dc}-{\text{BGL}}_t\right)/{\text{BGL}}_\text{dc}\times100$$

BGL_dc_ = Mean blood glucose level of diabetic control

BGL_t_ = Mean blood glucose level of treatment

Moreover, according to the 2020 edition of the AVMA Guidelines for Animal Euthanasia, the animals were compassionately euthanized while they were under general anesthesia [35]. All the mice were euthanized after the experiment through cervical dislocation while they were anesthetized with 3% sodium pentobarbital [35].

### In silico studies

#### Preparation of macromolecules

The selected receptors were acquired from the RCSB Protein Data Bank (https://www.rcsb.org; accessed on March 1, 2023) with specific PDB IDs (1EL3 for aldose reductase and 3BAJ for α-amylase). Before docking, any ligands and water molecules attached to the receptor's active site were eliminated.

#### Molecular docking

AutoDockTools (ADT) and AutoDock Vina programs [36] were utilized for molecular docking studies. To dock the compounds against the active site of the aldose reductase and α-amylase proteins (PDB ID: 1EL3 and PDB ID: 3BAJ, respectively), a standard protocol was followed. A grid box was created with dimensions of 18, 20, and 20 in the x, y, and z directions, respectively, and a spacing of 1.0 Å between grid points, where the center grid box coordinates were set to 17.56 Å, -7.646 Å, and 15.561 Å for the aldose reductase protein (PDB ID: 1EL3). Similarly, the grid box for the α-amylase proteins (PDB ID: 3BAJ) was built with different lengths in the x, y, and z directions, specifically 32, 30, and 24, respectively. The spacing between grid points was set to 1.0 Å. The central grid box had dimensions of 9.412 Å, 18.615 Å, and 43.422 Å. AutoDock Vina scoring functions were employed to rank the binding energies of nine conformations for each ligand. The most desirable conformations with the least free binding energy were selected to investigate how the target receptor and ligands interacted.

#### Prediction of activity spectra for substances (PASS) study

Every natural substance has different biological effects, which is a well-known fact. In order to predict the range of biological activity of synthetic compounds for the development of new drugs, the PASS study has proven to be an extremely useful instrument. The potential of PASS to forecast the pharmacological activity spectra of natural products is still unexplored [37]. The PASS online tool (www.way2drug.com/passonline/predict.php; accessed on March 30, 2023) was employed to examine the pass prediction to determine the isolated compounds' possible antidiabetic effects based on the molecular structure.

#### Prediction of pharmacokinetic (ADME) properties

Using the online server admetSAR (http:/Immd.ecust.edu.cn/admetsar2; accessed on March 30, 2023), the in silico pharmacokinetic properties, such as absorption, distribution, metabolism, and excretion (ADME) were predicted [38].

#### In silico toxicity prediction

The toxicological parameters of the isolated compounds were also projected by utilizing online tool ProTox II (https://tox-new.charite.de/protox_II/; accessed on March 30, 2023). In this study, we evaluated various properties of the isolated compounds, such as median lethal dose (LD_50_) (mg/kg) values, toxicity classification, as well as their potential impact on the liver (hepatotoxicity), ability to cause cancer (carcinogenicity), effect on the immune system (immunotoxicity), genetic mutability (mutagenicity), and ability to damage cells (cytotoxicity).

### Statistical analysis

The results obtained from statistical analysis are displayed as mean ± SD. In order to conduct the analysis, SPSS IBM (version 26) was used. In order to determine statistical significance, Dunnett's Multiple Comparison Test was run as a one-way analysis of variance (ANOVA), and p-values less than 0.05, 0.01, and 0.001 were considered as level of significance.

## Results

### Structure elucidation of the isolated compounds

The chloroform soluble partitionate was subjected to GPC over Sephadex LH-20, followed by preparative TLC and compound (1) and (2) were isolated as pure. Using ^1^H, ^13^C NMR, COSY, HSQC, HMBC, MS spectral data, these compounds were identified and characterized as 4΄-*O*-methyl (2″,4″-di-*E-p*-coumaroyl) afzelin (1) and quercetin 3-*O*-(2″,4″-di-*E*-*p*-coumaroyl)-α-L-rhamnopyranoside (2).

Compound (1) was obtained in the form of an amorphous powder that had a yellowish-white color. Upon examining it using ESI–MS, the pseudo-molecular ion peaks for [M + H]^+^ at m/z 739.000 and [M + Na]^+^ at m/z 761.100 were observed, suggesting that its molecular formula is C_40_H_34_O_14_, the spectrum is depicted in Supplementary Figure S1. The ESI–MS also exhibited a pseudo-molecular ion peak at *m/z* 737.400 in the negative mode (Supplementary Figure S2). The ^1^H and ^13^C NMR spectrum demonstrated a 4′-*O*-methyl kaempferol unit, a rhamnopyranosyl moiety and two *trans-p*-coumaroyl units at C-2″ and at C-4″ of the rhamnopyranosyl unit (Table 1). All the ^1^H-NMR and ^13^C-NMR spectra, including the HSQC, HMBC, and COSY spectra of the isolated compound 1 are available in the supplementary file (Supplementary Figures S3-S19).

**Table 1:** The spectral data for compound (1) and (2) in CD_3_OD obtained using nuclear magnetic resonance (NMR) techniques. The NMR measurements were performed at frequencies of 400 MHz and 100 MHz for proton (1H) and carbon (13C) nuclei, respectively

4ʹ-*O-*Methyl (2ʹʹ, 4ʹʹ-di-*E-p*-coumaroyl) afzelin (1)			Quercetin 3-*O*-(2ʹʹ,4ʹʹ-di-*E*-*p-*coumaroyl)-α-L-rhamnopyranoside (2)		
Position	**δC**	**δH, mult (*J* in Hz)**	**Position**	**δC**	**δH, mult (*J* in Hz)**
**2**	157.6, C		2	158.06, C	
**3**	133.7, C		3	132.41, C	
**4**	177.9, C		4	177.78, C	
**5**	161.9, C		5	176.83, C	
**6**	98.6, CH	6.21, s	6	99.00, CH	6.22, d (2.0)
**7**	164.7, C		7	-	
**8**	93.5, CH	6.39, br.s	8	93.55, CH	6.40, d (2.0)
**9**	157.3, C		9	157.25, C	
**10**	104.6, C		10	104.30, C	
**1ʹ**	122.4, C		1ʹ	121.43, C	
**2ʹ/6ʹ**	130.5, CH	7.91, d (8.4)	2ʹ	148.69, CH	7.33, dd (8.0, 2.0)
**3ʹ/5ʹ**	114.0, CH	7.18, d (8.4)	3ʹ	147.92, CH	7.01, d (8.0)
**4ʹ**	162.2, C		4ʹ	-	
**7ʹ**	54.7, CH_3_	3.86, s	6ʹ	-	7.40, d (2.0)
Rhamnosyl					
**1ʹʹ**	98.0, CH	5.70, br.s	1ʹʹ	99.04, CH	5.77, s
**2ʹʹ**	71.7, CH	5.52, m	2ʹʹ	71.74, CH	5.57, br.s
**3ʹʹ**	67.1, CH	4.15, m	3ʹʹ	67.08, CH	4.21, dd (9.6, 3.6)
**4ʹʹ**	73.3, CH	4.95, dd (10.0, 9.6)	4ʹʹ	73.40, CH	5.00, dd (10.0, 9.6)
**5ʹʹ**	68.4, CH	3.28, m	5ʹʹ	68.37, CH	3.32, m
**6ʹʹ**	16.3, CH_3_	0.83, d (6.4)	6ʹʹ	16.30, CH3	0.87, d (6.4)
Coumaroyl-A					
**1ʹʹʹ**	125.8, C		1ʹʹʹ	125.83, C	
**2ʹʹʹ/6ʹʹʹ**	129.9, CH	7.50, d (8.4)	2ʹʹʹ/6ʹʹʹ	129.97, CH	7.58, d (8.8)
**3ʹʹʹ/5ʹʹʹ**	114.5, CH	6.83, d (8.4)	3ʹʹʹ/5ʹʹʹ	115.43, CH	6.86, d (8.8)
**4ʹʹʹ**	160.0, C		4ʹʹʹ	159.97, C	
**7ʹʹʹ**	146.1, CH	7.68, d (16.0)	7ʹʹʹ	146.04, CH	7.71, d (16.0)
**8ʹʹʹ**	113.3, CH	6.40, d (16.0)	8ʹʹʹ	113.31, CH	6.45, d (15.8)
** > C = O**	166.8, C		> C = O	166.85, C	
Coumaroyl-B					
**1ʹʹʹʹ**	125.7, C		1ʹʹʹʹ	125.83, C	
**2ʹʹʹʹ/6ʹʹʹʹ**	130.0, CH	7.48, d (8.4)	2ʹʹʹʹ/6ʹʹʹʹ	129.97, CH	7.52, d (8.4)
**3ʹʹʹʹ/5ʹʹʹʹ**	115.5, CH	6.79, d (8.4)	3ʹʹʹʹ/5ʹʹʹʹ	115.43, CH	6.82, d (8.4)
**4ʹʹʹʹ**	160.1, C		4ʹʹʹʹ	161.87, C	
**7ʹʹʹʹ**	145.5, CH	7.55, d (16.0)	7ʹʹʹʹ	145.64, CH	7.62, d (16.0)
**8ʹʹʹʹ**	113.6, CH	6.26, d (16.0)	8ʹʹʹʹ	113.59, CH	6.34, d (15.8)
** > C = O**	167.0, C		> C = O	Not detected	

For the 4′-*O*-methyl kaempferol unit, the ^1^H NMR (400 MHz, CD_3_OD) spectrum displayed two singlets at δ 6.21 (1H, s, H-6) and 6.39 (1H, br. s, H-8), two doublets at δ 7.91 (2H, *J* = 8.4 Hz, H-2΄/6΄) and 7.18 (2H, *J* = 8.4 Hz, H-3΄/5΄) and a methoxy group at δ 3.86 (3H, s) for the C-4΄ position. Furthermore, signals at δ 5.70 (1H, br. s, H-1″), 5.52 (1H, m, H-2″), 4.15 (1H, m, H-3″), 4.95 (1H, dd, *J* = 10.0, 9.6 Hz, H-4″), 3.28 (1H, m, H-5″) and a doublet at δ 0.83 (1H, *J* = 6.4 Hz, H-6″) have led to the identification of a rhamnopyranosyl unit. The signals at δ 7.55 (1H, d, *J* = 16.0 Hz, H-7⁗), 6.26 (1H, d, *J* = 16.0 Hz, H-8⁗) and 7.68 (1H, d, *J* = 16.0 Hz, H-7‴) & 6.40 (1H, d, *J* = 16.0 Hz, H-8‴) indicated the existence of two trans-p-coumaroyl units with a trans-configuration. The aromatic ring protons for *p*-coumaroyl unit-A were identified by the doublets at δ 7.50 (2H, *J* = 8.4 Hz, H-2‴/6‴) and 6.83 (2H, *J* = 8.4 Hz, H-3‴/5‴) while the aromatic ring protons for p-coumaroyl unit-B were ascertained by the doublets at δ 7.48 (2H, *J* = 8.4 Hz, H-2⁗/6⁗) and 6.79 (2H, *J* = 8.4 Hz, H-3⁗/5⁗).

**Fig. 1:**
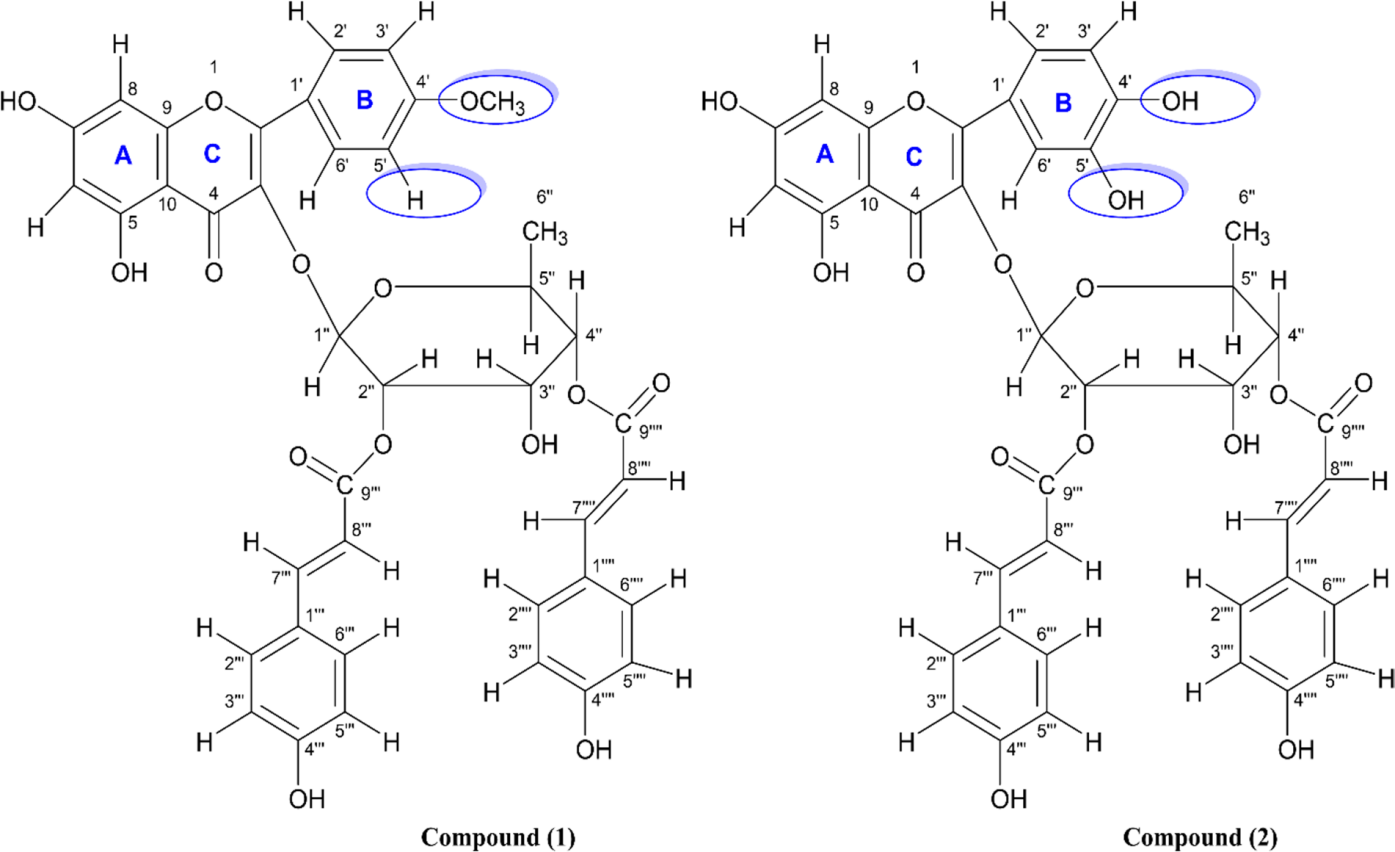
Chemical structures of 4΄-*O*-methyl (2″,4″-di-*E*-p-coumaroyl) afzelin (compound 1) and and its derivative quercetin 3-*O*-(2″,4″-di-*E*-p-coumaroyl)-α-L-rhamnopyranoside (compound 2) from the leaves of *L. glutinosa*

Furthermore, five sets of COSY correlations between H-1'' and H-2'' at δ 5.70 and 5.52, between H-2'' and H-3'' at δ 5.52 and 4.15, between H-3'' and H-4'' at δ 4.15 and 4.95, between H-4'' and H-5'' at δ 4.95 and 3.28, between H-5'' and H-6'' at δ 3.28 and 0.83, revealed the presence of a rhamnopyranosyl moiety in compound (1). The COSY NMR spectral data demonstrated correlations between H-7'''' and H8'''' at δ 7.55 and 6.21, H-7''' and H-8''' at δ 7.68 and 6.40, between H-3'''/5''' and H2'''/6''' at δ 7.50 and 6.83, between H-3''''/5'''' and H-2''''/6'''' at δ 7.48 and 6.79 confirmed the presence of two coumaroyl units in compound (1) isolated from *L. glutinosa* (Fig. 1)*.*

The HSQC spectral data provided important information to reveal the structure. In the case of rhamnopyranosyl moiety, the HSQC spectrum showed cross peaks for H2''- C2'' at δ_H_ 5.52/ δ_C_ 71.7, H4''- C4'' at δ_H_ 4.95/ δ_C_ 73.3, H3''- C3'' at δ_H_ 4.15/ δ_C_ 67.0, interactions for H5''- C5'' observed at δ_H_ 3.28/ δ_C_ 68.4 and H6''- C6'' at δ_H_ 0.83/ δ_C_ 16.3 for the rhamnopyranosyl moiety in compound (1).

**Fig. 2:**
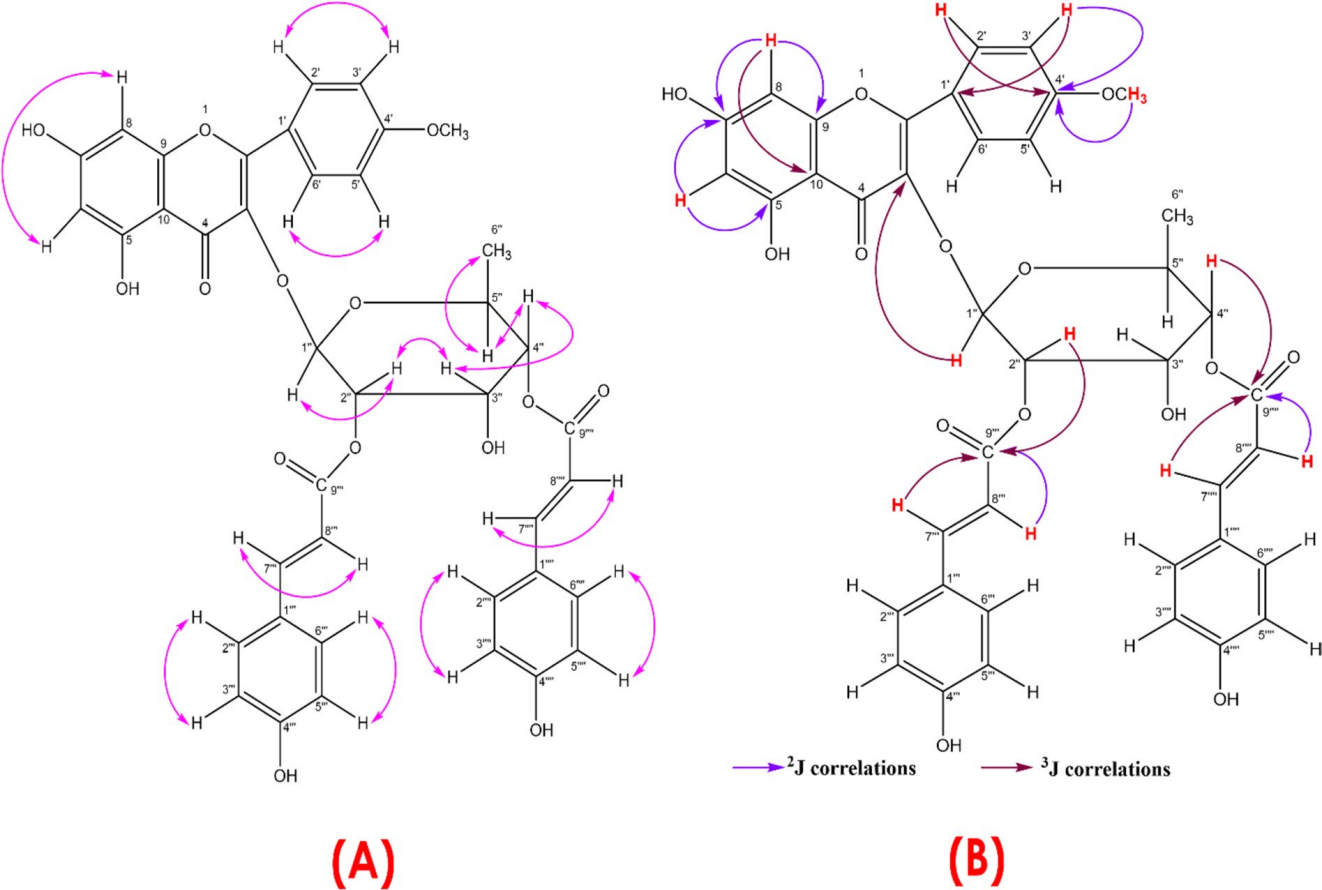
Key COSY correlations (**A**) and key HMBC correlations (**B**) observed for compound 1

The HMBC correlations from δ_H_ 7.91 (H-2΄/6΄) to δ_C_ 157.6 (C-2) as well as δ_H_ 3.46 (OCH_3_) to δ_C_ 162.2 (C-4΄) ascribed the presence of kaempheride skeleton. The linkage between C-1'' of the rhamnopyranosyl group and C-3 of the kaempherol unit was suggested by the correlation observed between δH 5.70 (H-1'') and δC 133.7 (C-3). Additionally, the presence of two trans-p-coumaroyl groups at C-2'' and C-4'' of the rhamnopyranosyl unit was confirmed by HMBC correlations from δH 5.52 (H-2'') to δC 166.9 (> C = O) and from δH 4.95 (H-4'') to δC 167.0 (> C = O). The connections between the 4′-*O*-methyl kaempferol unit with the rhamnopyranosyl unit as well as between the rhamnopyranosyl unit with two trans-p-coumaroyl units were established by HMBC correlations as depicted in Fig. 2. Thus, compound (1) was identified as 4΄-*O-*methyl-(2″,4″-di-*E*-*p*-coumaroyl) afzelin. To confirm the identification of compound 1, its 1H, 13C, and mass spectral data were compared with the data published by Huang et al. [39] and Li et al. [5]. This comparison further supported the identification of compound 1.

Compound (2) was isolated as a yellowish white powder. The ESI–MS spectrum of the compound showed a pseudo-molecular ion peak [M + Na]^+^ at *m/z* 763.007 which indicated a molecular formula of C_39_H_32_O_15_ (Supplementary Fig. 20). This was also further supported by a [M-H]^+^ peak at m/z 739.189 in negative mode ESI–MS spectrum (Supplementary Fig. 21). The ^1^H NMR (400 MHz) spectra of compounds 1 and 2 were almost identical, which suggested that compound (2) is a derivative of the compound 1. The B-ring protons in compound 1 were evident as an AA΄ and BB΄ pattern, whereas, in compound 2 the trisubstituted B-ring protons appeared as an *ortho* (δ 7.01, *J* = 8.0 Hz), *ortho-meta* (δ 7.33, *J* = 8.0, 2.0 Hz) and *meta* (δ 7.40, *J* = 2.0 Hz) coupled protons. In addition, the methoxyl group signal observed for compound (1) could not be seen in ^1^H NMR spectrum of compound 2. This indicated that in the latter one, the hydroxyl group replaced the methoxyl group. The presence of only B-ring proton signals and lack of methoxyl group resonance also ascertained that the remaining carbon has hydroxyl moiety. All the ^1^H-NMR and ^13^C-NMR spectra, including the HSQC, HMBC, and COSY spectra of the isolated compound 2 are available in the supplementary file (Supplementary Figures S22-S31).

The ^1^H NMR (400 MHz) spectrum demonstrated signals assignable to a 4′,5′-dihydroquercetin unit, a rhamnopyranosyl moiety and two *trans-p*-coumaroyl units at C-2″ and at C-4″ of the rhamnopyranosyl unit. The ^1^H NMR spectrum (Table 1) displayed resonances for the 4′,5′-dihydroquercetin moiety as four doublets at δ 6.22 (1H, *J* = 2.0 Hz, H-6), 6.40 (1H, *J* = 2.0 Hz, H-8), 7.01 (1H, *J* = 8.0 Hz, H-3΄), and 7.40 (1H, *J* = 2.0 Hz, H-6΄) and a double-doubles at δ 7.33 (1H, *J* = 8.0, 2.0 Hz, H-2΄). Furthermore, it was determined that there is a rhamnopyranosyl component present based on the signals detected at δ 5.77 (1H, s, H-1″), 5.57 (1H, br. s, H-2″), 4.21 (1H, dd, *J* = 9.6, 3.6 Hz, H-3″), 4.95 (1H, dd, *J* = 10.0, 9.6 Hz, H-4″), 3.32 (m, H-5″), and 0.87 (d, *J* = 6.4 Hz, H-6″) (Table 1). Two p-coumaroyl units with trans-configuration were clearly evident from signals at δ 7.62 (1H, d, *J* = 16.0 Hz, H-7⁗), 6.34 (1H, d, *J* = 15.8 Hz, H-8⁗), 7.71 (1H, d, *J* = 16 Hz, H-7‴), and 6.45 (1H, d, *J* = 15.8 Hz, H-8‴) as demonstrated in Table 1.

**Fig. 3:**
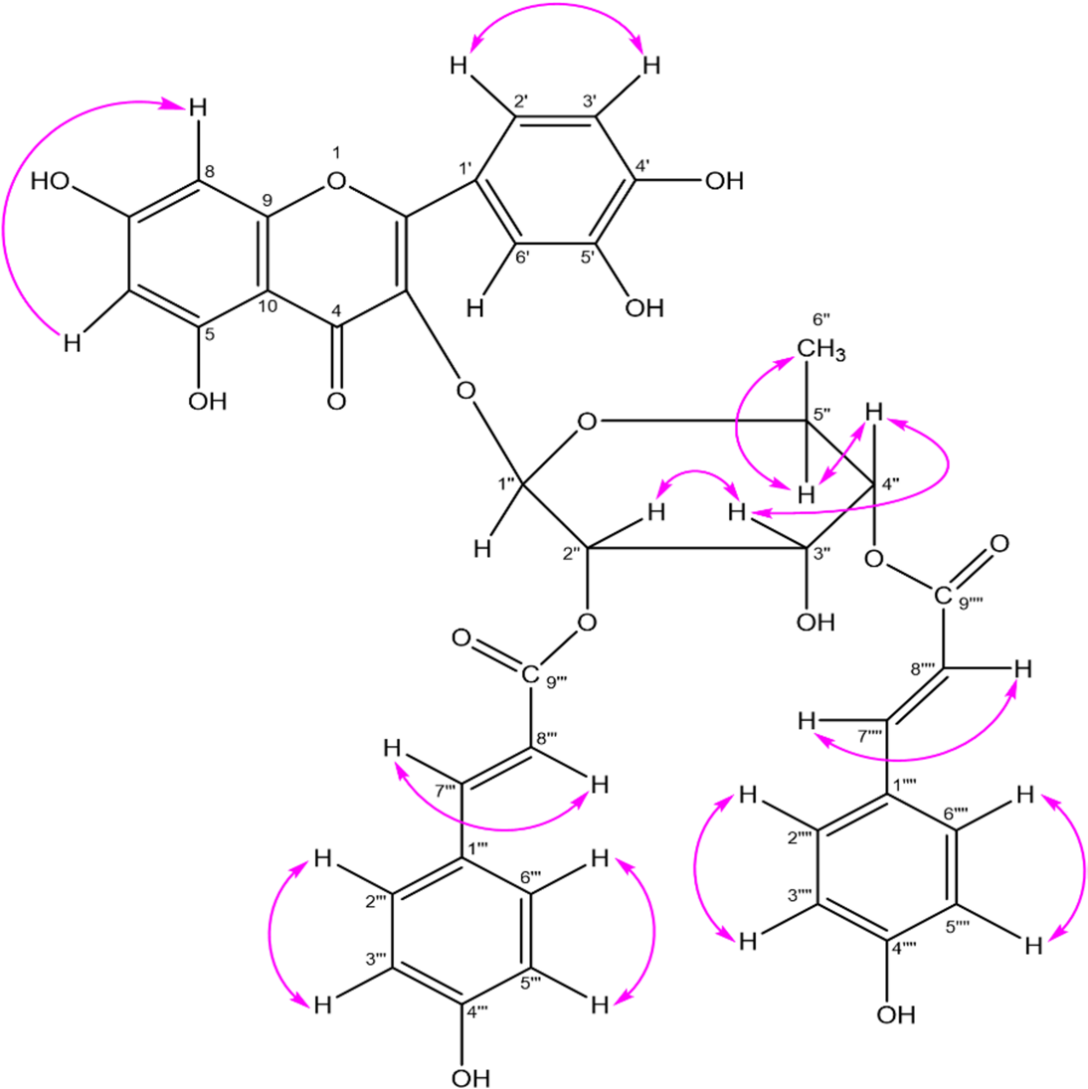
Key COSY correlations observed for compound 2

The COSY NMR spectrum exerted the expected correlations between H-2'' and H-3'' at δ 5.57 and 4.21, between H-3'' and H-4'' at δ 4.21 and 4.95, between H-4'' and H-5'' at δ 5.00 and 3.32 and between H-5'' and H_3_-6'' at δ 3.32 and 0.87. These five correlations demonstrated the presence of a rhamnopyranosyl moiety in LGC-45–3. Furthermore, COSY correlations between H-7'''' and H- 8'''' at δ 7.62 and 6.74, between H-7''' and H-8''' at δ 7.71 and 6.45, between H-3'''/5''' and H-2'''/6''' at δ 7.52 and 6.82, between H-3''''/5'''' and H-2''''/6'''' at δ 7.58 and 6.86 confirmed the presence of two coumaroyl units in compound 2 (Fig. 3).

The assignment for compound 2 has been done based on its ^1^H NMR and ^13^C NMR spectral data and comparison with the closely related compound 1. Thus, compound 2 was identified as quercetin-3-*O*-(2ʹʹ,4ʹʹ-di-*E*-*p*-coumaroyl)-α-L-rhamnopyranoside. It can also be named as 5΄-hydroxyl-(2″,4″-di-*E-p*-coumaroyl) afzelin. Although the compound 2 had been documented in *Machilus litseifolia* [5] and *Mammea longifolia* [40], this is the initial account of its presence in *Litsea* species.

### Antidiabetic activity

The effectiveness of the methanol extract of *L. glutinosa* (MELG) in treating diabetes was tested on Swiss Albino mice (Supplementary Figure S32). The mice were given STZ at multiple low doses (40 mg/kg/day) for five consecutive days, which caused their blood glucose levels to rise significantly. There was a notable rise in the blood glucose levels of the diabetic control group, which did not receive any treatment. By the 7th day of treatment, the blood glucose level was recorded to be 16.3 ± 3.15 mmol/L for the untreated diabetic control group. The study involved several groups, including a control group, an untreated diabetic control group, a treated group with standard medication, and two groups treated with different doses of extracts. The blood glucose levels of all groups were measured on the third, fifth, and seventh days. It was found that administering the usual dose of metformin, which is 50 mg/kg of body weight, led to a significant decrease in blood sugar levels (*p* < 0.01) compared to the group that didn't receive any treatment. The researchers calculated that the standard medication reduced the risk of diabetes by 73.83%, as shown in Table 2. The administration of MELG at a dosage of 300 mg/kg bw resulted in a significant (*p* < 0.05) decrease in blood glucose levels on the seventh day. Furthermore, when the dosage was 500 mg/kg bw, the reduction in blood glucose levels was even more significant (*p* < 0.001) after a seven-day treatment with MELG. The MELG reduced blood glucose levels by a percentage like that of the standard metformin. Specifically, at a dose of 500 mg/kg b.w./day, the percentage inhibition was 66.69%, which is comparable to the level seen in the diabetic control group that was treated with metformin (Table 2).

**Table 2:** Antidiabetic effect of methanol extract of *L. glutinosa* (MELG) on STZ induced diabetic mice

**Groups**	**Oral Dose (mg/kg b.w./day)**	**Blood glucose level (mmol/liter)**				**% inhibition of blood glucose level**
		**1st Day**	**3rd Day**	**5th Day**	**7th Day**	
Control (non-diabetic)	-	5.20 ± 0.17	5.01 ± 0.13	5.50 ± 0.35	4.87 ± 0.26	-
Untreated diabetic control	-	8.0 ± 0.53	9.0 ± 0.71	13.6 ± 1.02	16.3 ± 3.15	-
Metformin HCl	50	12.46 ± 0.67	5.53 ± 0.27**	4.46 ± 0.14**	4.26 ± 0.32**	73.87%
MELG-1	300	10.2 ± 1.02	8.02 ± 0.12	7.53 ± 0.32	6.0 ± 0.12*	63.19%
MELG-2	500	11.2 ± 0.82	7.22 ± 0.52	6.22 ± 0.32*	5.43 ± 0.12**	66.69%

The data is presented using the format as mean ± standard deviation (SD), with a sample size of 5. Statistical significance is denoted by the levels of probability indicated by *p < 0.05 or **p < 0.01, when compared to the diabetic control group

### Inhibition of aldose reductase

**Fig. 4:**
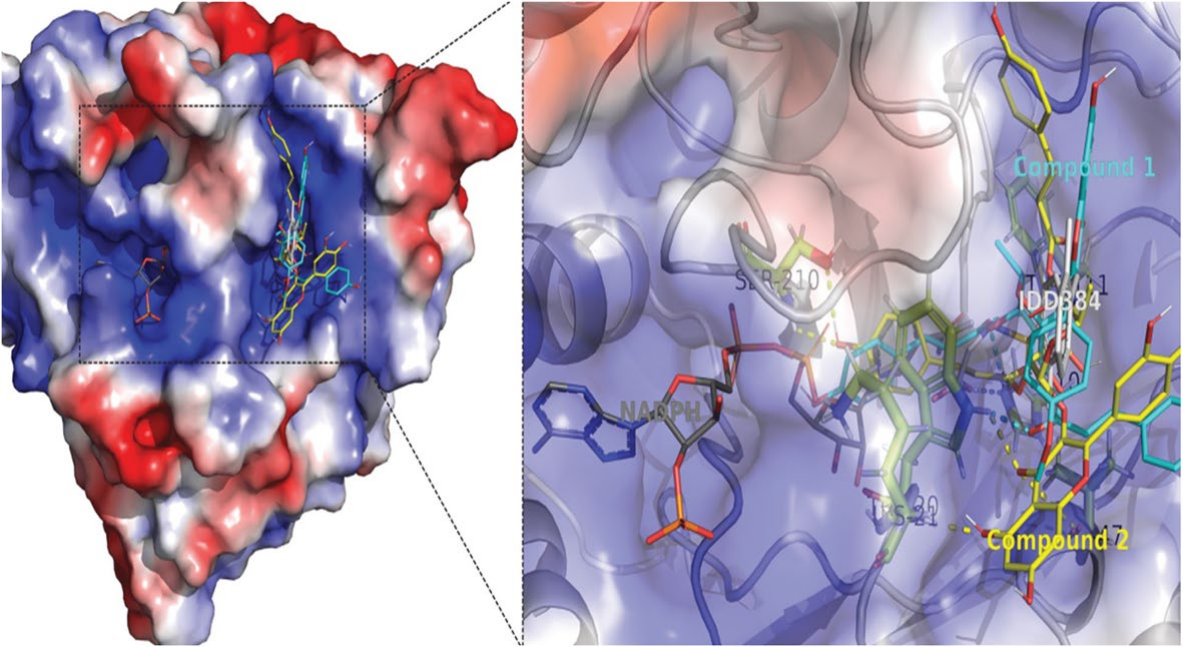
The superposition of the three compounds: Compound 1 represented in cyan sticks, compound 2 in yellow sticks, and the standard inhibitor IDD384 (PDB ID: 1EL3) in white sticks. These compounds are bound to the active site of the human aldose reductase protein structure, as depicted by the electrostatic surface potential representation. The electrostatic surface potential was determined using the APBS server [42], and the accessible surface area has been color-coded according to the calculated electrostatic potential. The colors range from red (representing a potential of -3 kT/e) to blue (representing a potential of 3 kT/e)

Molecular docking analysis of the isolated compounds 1 and 2 showed promising docking scores within the active site of human aldose reductase (AR2; PDB ID: 1EL3) (Fig. 4). Compounds 1 and 2 showed better binding affinities (− 10.3, and − 11.0 kcal/mol, respectively) compared to that of the established inhibitor IDD384 [41], which was found to be − 9.5 kcal/mol. Compound 1 formed polar contacts with Trp-20, His-110, and Trp-111 residues and compound 2 formed polar contacts with Trp-20, Lys-21, Val-47, and Ser-210 residues (Table 3).

**Table 3:** Binding affinities (kcal/mol) obtained from molecular docking of compound 1 and 2 against human aldose reductase (AR2; PDB ID: 1EL3) and α-amylase (PDB ID: 3BAJ) proteins

Proteins	Compounds	Binding affinity (kcal/mol)	Polar contact forming residues
human aldose reductase (AR2; PDB ID: 1EL3)	Compound **1**	− 10.3	Trp-20, His-110, and Trp-111
	Compound **2**	− 11.0	Trp-20, Lys-21, Val-47, and Ser-210
α-amylase (PDB ID: 3BAJ)	Compound **1**	− 9.4	Tyr-151, Thr-163, Arg-195, Asp-197, His-201, and His-299
	Compound **2**	− 8.9	Gln-63, Arg-195, Asp-197, and His-299

Compounds 1 and 2 showed inhibition constants (K*i*) of 27 nM and 8 nM, respectively, which were calculated using the binding energies (ΔG) and the formula K*i* = exp(ΔG/RT), the given statement defines the variables as follows: R is the gas constant that applies universally, and its value is 1.985 × 10^–3^ kcal mol^−1^ K^−1^. T denotes temperature and is set at 298.15 K. These findings suggest that both of the isolated compounds might be promising inhibitors of human aldose reductase.

### Inhibition of α-amylase

**Fig. 5:**
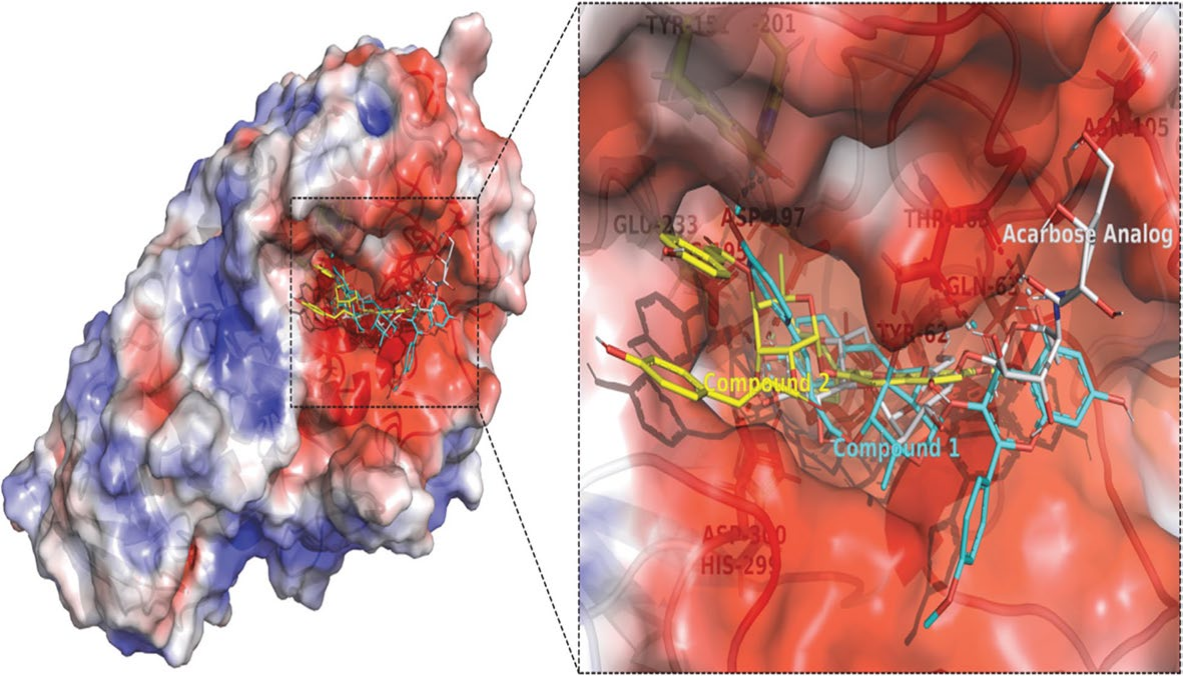
Representation of the superimposed structures of compound 1 (cyan stick), compound 2 (yellow stick), and the standard inhibitor acarbose analog (white stick; PDB ID: 3BAJ). The active site of the human pancreatic α-amylase protein structure was shown using an electrostatic surface potential representation. This was achieved by calculating the electrostatic potential using the APBS server and coloring the accessible surface area based on the potential from − 3 kT/e (red) to 3 kT/e (blue)

Molecular docking analysis of the isolated compounds 1 and 2 showed better docking scores within the active site of human pancreatic α-amylase (PDB ID: 3BAJ) (Fig. 5). Compounds 1 and 2 showed better binding affinities (− 9.4, and − 8.9 kcal/mol, respectively) compared to that of the established inhibitor acarbose [43], which was found to be -7.6 kcal/mol. Compound 1 formed polar contacts with Tyr-151, Thr-163, Arg-195, Asp-197, His-201, and His-299 residues and compound 2 formed polar contacts with Gln-63, Arg-195, Asp-197, and His-299 residues (Table 3).

The inhibition constants (K*i*) of the compound 1 and 2 (124, and 290 nM, respectively) were obtained from the binding energies (ΔG) using the formula: K*i* = exp(ΔG/RT), the expression "R" represents the gas constant applicable universally, and its value is 1.985 × 10^–3^ kcal mol^−1^ K^−1^. "T" on the other hand, refers to the temperature and has a fixed value of 298.15 K. These findings suggest that both isolated compounds 1 and 2 might be promising inhibitors of human pancreatic α-amylase.

### PASS analysis

The PASS user receives a list of anticipated activity types and their corresponding likelihood of being "active" (Pa) or "inactive" (Pi), with probabilities ranging from zero to one. The compound is expected to only expose activities with a Pa value greater than Pi. In case the Pa value exceeds 0.3, it is probable that the compound will disclose the activity in experimental conditions [44]. The antidiabetic, aldose reductase inhibitory, and α-glucosidase inhibitory activities were listed in Table 4 based on their Pa and Pi values. Both compounds exerted higher Pa values than Pi values.

**Table 4:** The anticipated specific pharmacological effects of the isolated compounds obtained from PASS prediction

Pharmacological activities	Probability parameters	Compound 1	Compound 2
Antidiabetic activity	Pa	0.330	0.337
	Pi	0.067	0.065
Aldose reductase inhibitor	Pa	0.593	0.642
	Pi	0.002	0.002
α-glucosidase inhibitor	Pa	0.350	0.471
	Pi	0.003	0.002

### Prediction of ADME

The various properties of the isolated compounds, for example, human intestinal absorption (HIA), Caco-2 permeability, cytochrome P450 enzyme inhibition level, and P glycoprotein inhibitor (PGI) were considered for the analysis. Table 5 contains a summary of the results that were acquired. It is worth mentioning that both Compounds 1 and 2 displayed exceptional absorption in the human intestine and did not have any inhibitory effects on cytochrome P450. Furthermore, these compounds demonstrated excellent volume of distribution and renal clearance.

**Table 5:** ADME profiles of the Compound 1 and 2

Categories	Properties	Value	Interpretation	Value	Interpretation	Explanation
		**Compound 1**		**Compound 2**		
**Absorption**	P-glycoprotein (P-gp)-inhibitor	0.598	Medium	0.051	Excellent	Probability of being Pgp-inhibitor; 0–0.3: excellent; 0.3–0.7: medium
	Pgp-substrate	0.031	Excellent	0.096	Excellent	Probability of being Pgp-substrate; 0–0.3: excellent; 0.3–0.7: medium
	Human intestinal absorption (HIA)	0.091	Excellent	0.129	Excellent	Probability of having HIA < 30%
	Caco-2 Permeability	-5.534	Optimal	-5.893	Optimal	Excellent: > -5.15log cm/s
	Madin − Darby Canine Kidney cells (MDCK) Permeability	4.17 × 10^–5^	Excellent	2.78 × 10^–5^	Excellent	Excellent: > 2 × 10^−6^ cm/s
**Distribution**	Blood–brain barrier (BBB) penetration	0.007	Excellent	0.007	Excellent	Unit of BBB penetration is cm/s
	Volume of distribution	0.502	Excellent	0.477	Excellent	Optimal: 0.04–20 L/kg
**Metabolism**	CYP 1A2 inhibitor	0.501	-	0.628	-	Probability of being inhibitor
	CYP 1A2 substrate	0.061	-	0.024	-	Probability of being substrate
	CYP 2C19 inhibitor	0.917	-	0.675	-	Probability of being inhibitor
	CYP 2C19 substrate	0.048	-	0.038	-	Probability of being substrate
	CYP 2C9 inhibitor	0.605	-	0.552	-	Probability of being inhibitor
	CYP 2C9 substrate	0.976	-	0.954	-	Probability of being substrate
	CYP 2D6 inhibitor	0.912	-	0.73	-	Probability of being inhibitor
	CYP 2D6 substrate	0.841	-	0.293	-	Probability of being substrate
	CYP 3A4 inhibitor	0.568	-	0.382	-	Probability of being inhibitor
	CYP 3A4 substrate	0.107	-	0.064	-	Probability of being substrate
**Excretion**	Clearance	5.913	Excellent	6.436	Excellent	Moderate: 5–15 mL/min/kg
	Half-life	0.776	Poor	0.904	Poor	Probability of having long half-life

*HIA* Human intestinal absorption, *PGI* P-glycoprotein inhibitor

### In silico toxicity prediction

Table 6 presents data on seven calculated parameters, viz., LD_50_, toxicity classification, and the likelihood of inducing hepatotoxicity, carcinogenicity, immunotoxicity, mutagenicity, and cytotoxicity of the both isolated molecules. LD_50_ (mg/kg) refers to the median lethal dose that results in the death of 50% of the experimental subjects when exposed to a particular compound. All the cases, the compounds 1 and 2 exerted inactive except the Immunotoxicity.

**Table 6:** Toxicity prediction of the compounds 1 and 2

Parameters	Compound 1	Compound 2
LD_50_ (mg/Kg)	5000 mg/kg	5000 mg/kg
Class	V	V
Hepatotoxicity	-	-
Carcinogenicity	-	-
Immunotoxicity	+	+
Mutagenicity	-	-
Cytotoxicity	-	-

“ + ” and “-” mean active and inactive, respectively

## Discussion

Since ancient times, medicinal plants have been used as traditional medicine. Still, due to their protective effects and other beneficial qualities for human health, these plants continue to attract people, particularly in developing nations, to use them to treat common ailments [45–48]. According to a report of WHO, over 80% of the global population relies on herbal remedies for their basic medical needs, and 21,000 plant species may be used as medicinal plants [49]. Many plant-derived constituents serve potential responses as therapeutic agents with various pharmacological actions, including anticancer, nephroprotective, hepatoprotective, neuroprotective, and antidiabetic properties [50–53]. In order to examine medicinal plants' therapeutic potential, several procedures are needed, including identifying and characterizing bioactive compounds [54]. Indigenous people utilize medicinal plants in a variety of ways, such as pastes, juices, or boiled leaf extracts, even without a clear understanding of the ideal dose profile [49]. This stimulates the interest of researchers who want to learn more about the pharmacologic potentiality of the effective solvent extraction technique. Methanol is frequently employed in the extraction of medicinal plants like *L. glutinosa* due to its high polarity index, which allows bioactive substances such as flavonoids, tannins, terpenoids, and alkaloids to mix with it rapidly. Consequently, the current study aimed to isolate the potent phytochemicals and evaluate the pharmacological activates of leaves of *L. glutinosa*, focusing on antidiabetic activity via in vivo and in silico techniques, i.e., software-based molecular docking and online server-based ADMET prediction.

The present study demonstrated the identification of two new flavonoid glycosides from the leaves of *L. glutinosa* as 4΄-O-methyl (2″,4″-di-*E*-p-coumaroyl) afzelin (1) and quercetin 3-O-(2″,4″-di-*E*-p-coumaroyl)-α-L-rhamnopyranoside (2), which were characterized and confirmed by ^1^H and ^13^C NMR, COSY, HSQC, HMBC spectral data. Previously, compound 1 was reported from *Machilis litseifolia* [5] and *Lindera akoensis* [39]. Compound 2 was reported from *Machilis litseifolia* [5] and *Mammea longifolia* [40]. However, according to our knowledge, this is the first report where both phytochemicals have ever been isolated from *Litsea* species.

Elevated and fluctuating blood glucose levels are a hallmark of diabetes which is recognized as a chronic multifactorial disease. The primary causes of high blood sugar in individuals with diabetes are reduced insulin secretion by the beta-cells in the pancreas and reduced insulin sensitivity in the liver and muscle cells. Chronic hyperglycemia in diabetics results in micro- and macrovascular consequences, including cardiovascular disorders, diabetic retinopathy, neuropathy, and nephropathy [3, 5]. *L. glutinosa* is a traditional herbal remedy that is frequently used to treat diarrhea and dysentery [55]. It is demonstrated though the current research that the methanolic leaf extract from the *L. glutonosa* plant species had a considerable impact in reducing diabetes in mice that had been induced with STZ.

In this study, rats were given repeated low doses of STZ, a highly selective pancreatic islet β-cell cytotoxic agent, to induce diabetes [32]. The pancreatic islets are partially damaged by this multiple, low-dose STZ, which sets off an inflammatory cascade that further reduces β-cell activity and ultimately leads to hyperglycemia and insulin insufficiency [32]. The current study found that MELG at a dose of 300 mg/kg b.w./day showed a significant (*p* < 0.05) reduction in fasting blood glucose levels on the 7th day. Similarly, the 500 mg/kg b.w./day dose of MELG significantly reduced blood glucose levels at the 5th and 7th days compared with the control group. Furthermore, inhibition of blood glucose level for the methanolic extract of the plant species at the dose of 500 mg/kg b.w./day was comparable with that of standard metformin (66.69% vs. 73.87%), which is considered as first-line oral antidiabetic drug. Metformin precisely lowers hepatic gluconeogenesis without boosting insulin secretion, lead to weight gain, or raise the risk of hypoglycemia [56]. Our findings indicated that oral administration of MELG (500 mg/kg b.w./day) had blood glucose-lowering effects that were nearly identical to those of metformin (50 mg/kg b.w./day). According to our searching experience, no study showed the in vivo glucose-lowering capacity of the leaf extract of *L. glutinosa*. However, the alkaloid-rich bark extract (50, 100, and 200 mg/kg) of the plant species exhibited adequate antidiabetic effects by reducing insulin resistance [23].

The current phytochemical analysis demonstrated the isolation of two flavonoid glycosides (compounds 1 and 2) which have exerted human aldose reductase inhibition and α-amylase inhibition capacity in computational docking study. The both isolated compounds 1 and 2 showed higher binding affinity towards the human aldose reductase (PDB ID: 1EL3) with docking scores − 10.3, and − 11.0 kcal/mol, respectively compared to the docking score (− 9.5 kcal/mol) of the established inhibitor IDD384 [41]. Similarly, the both candidates (compounds 1 and 2) showed better docking scores within the active site of human pancreatic α-amylase (PDB ID: 3BAJ) with binding affinities − 9.4 and − 8.9 kcal/mol, respectively compared to the docking score − 7.6 kcal/mol of the established inhibitor acarbose [43].

By blocking the enzymes α-amylase, α-glucosidase, and human aldose reductase, flavonoid glycosides have shown to have anti-diabetic activities in several prior investigations [5, 57–59]. Due to the rarity of these two isolated flavonoid glucosides (compounds 1 and 2) and the lack of available experimental data, it is possible to infer their specific mode of action against diabetes from the actions of their analogues. Li et al. [5] reported that 4΄-*O*-methyl (2″,4″-di-*E*-p-coumaroyl) afzelin (compound 1) and its three more analogues exerted IC_50_ values 5.9 to 35.3 μM, which is 8 to 91 times more potent than the standard drug acarbose (IC_50_ = 266.1 μM) against the α-glucosidase enzyme, which is associated with carbohydrate digestion in small intestine. Thus, the flavonoid glycosides prevent carbohydrates from being cleaved, reducing glucose absorption and lowering postprandial glycemic levels [3].

Moreover, both compounds exerted promising interaction profile with human aldose reductase enzyme, which is a major enzyme responsible for conversion of glucose to sorbitol in polyol pathway by reducing process through the oxidation of NADPH to NADP^+^ [60]. In hyperglycemic conditions, the overexpression of aldose reductase causes glucose to be converted into sorbitol at a rate two to four times faster than usual. This excess sorbitol accumulation in the body tissues triggers cell swelling due to changes in osmotic pressure, alterations in cell membrane function, and elevated oxidative stress. These detrimental cellular processes are closely linked to the development of long-term diabetic complications, including the formation of cataracts, retinopathy, neuropathy, and nephropathy [60, 61]. In addition, the increased expression of aldose reductase causes the histone deacetylase 3 (HDAC3) gene to decline and peroxisome proliferator-activated receptor gamma (PPARγ) signaling to increase. This causes an excessive fat accumulation of heart. Furthermore, several additional enzymes and diabetes-related indicators are inhibited by the overexpression of aldose reductase [61]. As a result, the isolated compounds 1 and 2 may stop or slow down the development of these long-term diabetic problems by blocking aldose reductase enzyme. These compounds bound with amino acid residues in aldose reductase, blocking the active site and impairing the enzyme's capacity to process substrate and generate products. As a result, aldose reductase inhibitors have become more significant in the therapeutic arena since they may be able to address long-term issues resulting from diabetes [61]. Furthermore, inhibiting α-amylase by these flavonoid glycosides might be another pathway of diabetes treatment. The α-amylase enzyme is primarily found in saliva and pancreatic juice and significantly increases blood sugar levels by breaking down starch and glycogen. These two isolated compounds may therefore show antidiabetic effects by inhibiting α-amylase [3]. Additionally, according to the in-silico PASS analysis, both compounds that demonstrated higher values of reliable activity (Pa) compared to reliable inactivity (Pi) were deemed appropriate for the intended antidiabetic, human aldose inhibitory, and α-glucosidase inhibitory activities. This finding is consistent with the results of the molecular docking study.

Moreover, the present investigation revealed that the isolated substances possess favorable pharmacokinetics and do not cause immediate oral toxicity. The classification and labeling of chemicals can be demonstrated as per the Globally Harmonized System of Classification and Labeling of Chemicals. This system categorizes chemicals into six classes based on their potential harm when swallowed. Class I includes chemicals that can be fatal if ingested and have an LD_50_ value of 5 or less. Class II comprises deadly chemicals if swallowed, but their LD_50_ value is between 5 and 50. Class III includes toxic chemicals, if ingested, with an LD_50_ value ranging from 50 to 300. If swallowed and have an LD_50_ value between 300 and 2000, harmful chemicals belong to Class IV. Class V consists of chemicals that may be harmful if ingested and have an LD_50_ value ranging from 2000 to 5000. Finally, Class VI includes non-toxic chemicals that have an LD_50_ value greater than 5000 [62, 63]. The both isolated compounds 1 and 2 belonged to toxicity class V having LD_50_ = 5000 mg/kg revealing the certain safety profile of the compounds. Furthermore, our prior research found that the crude methanolic leaf extract of this plant species exhibited a wider safety range, as indicated by an LD_50_ value exceeding 2000 mg/kg in an acute oral toxicity assessment [64].

### Study limitations and future research

Although the current study has several advantages, it also has a few shortcomings. One of these is that the study did not perform an extensive analysis of the phytochemicals from the plant species. Additionally, the study relied solely on in silico methods to predict the pharmacological effects of the isolated compounds without any experimental validation. Although the leaf extract of the plant species was studied by employing an acute toxicity test in our previous study [64] to determine its LD_50_ value, sub-acute, sub-chronic, and chronic toxicity assessments should be conducted in future studies to confirm the long-term safety profile after repeated intake of low doses of the plant species.

## Conclusions

The current study is going to report two flavonoid glucosides for the first time from *Litsea* species and they are 4΄-*O*-methyl-(2″,4″,-di-*E*-p-coumaroyl)-afzelin (compound 1) and quercetin-3-*O*-(2″,4″,-di-*E*-*p*-coumaroyl)-α-L-rhamno-pyranoside [or, 5΄-hydroxyl-(2″,4″-di-*E-p*-coumaroyl) afzelin] (compound 2). During the hypoglycemic activity test, it was observed that the level of blood glucose in mice with STZ-induced diabetes significantly decreased after being treated with the methanol extract of *L. glutinosa* for seven days. This indicates that the plant has encouraging potential as an antidiabetic agent. Furthermore, the outcomes of molecular docking study endorsed that the two individual substances could be effective inhibitors of human pancreatic α-amylase and aldose reductase. In addition, the in silico ADMET properties of compounds 1 and 2 were predominantly positive, which justifies selecting these compounds as primary candidates for future more extensive in vivo study and clinical investigation.

### Supplementary Information

**Additional file 1: Figure S1. **ESI-MS spectrum of 4΄-O-methyl (2″,4″-di-*E*-p-coumaroyl) afzelin (compound 1). **Figure S2.** ESI-MS spectrum of 4΄-O-methyl (2″,4″-di-*E*-p-coumaroyl) afzelin (compound 1). **Figure S3. **^1^H-NMR spectrum (400 MHz, CD_3_OD) of 4΄-O-methyl (2″,4″-di-*E*-p-coumaroyl) afzelin (compound 1). **Figure S4. **^1^H-NMR spectrum (400 MHz, CD_3_OD) of 4΄-O-methyl (2″,4″-di-*E*-p-coumaroyl) afzelin (compound 1) (expanded). **Figure S5.** 1H-NMR spectrum (400 MHz, CD_3_OD) of 4΄-O-methyl (2″,4″-di-*E*-p-coumaroyl) afzelin (compound 1) (expanded). **Figure S6.** ^1^H-NMR spectrum (400 MHz, CD_3_OD) of 4΄-O-methyl (2″,4″-di-*E*-p-coumaroyl) afzelin (compound 1) (expanded). **Figure S7.** ^1^H-NMR spectrum (400 MHz, CD_3_OD) of 4΄-O-methyl (2″,4″-di-*E*-p-coumaroyl) afzelin (compound 1) (expanded).** Figure S8. **^13^C-NMR spectrum (100 MHz, CD_3_OD) of 4΄-O-methyl (2″,4″-di-*E*-p-coumaroyl) afzelin (compound 1). **Figure S9. **^13^C-NMR spectrum (100 MHz, CD_3_OD) of 4΄-O-methyl (2″,4″-di-E-p-coumaroyl) afzelin (compound 1) (expanded). **Figure S10. **^13^C-NMR spectrum (100 MHz, CD_3_OD) of 4΄-O-methyl (2″,4″-di-*E*-p-coumaroyl) afzelin (compound 1) (expanded). **Figure S11. **^13^C-NMR spectrum (100 MHz, CD_3_OD) of 4΄-O-methyl (2″,4″-di-*E*-p-coumaroyl) afzelin (compound 1) (expanded). **Figure S12.** HSQC spectrum (400 MHz, CD_3_OD) of 4΄-O-methyl (2″,4″-di-*E*-p-coumaroyl) afzelin (compound 1). **Figure S13.** HSQC spectrum (400 MHz, CD_3_OD) of 4΄-O-methyl (2″,4″-di-*E*-p-coumaroyl) afzelin (compound 1) (expanded). **Figure S14.** HSQC spectrum (400 MHz, CD_3_OD) of 4΄-O-methyl (2″,4″-di-*E*-p-coumaroyl) afzelin (compound 1) (expanded). **Figure S15.** HMBC spectrum (400 MHz, CD_3_OD) of 4΄-O-methyl (2″,4″-di-*E*-p-coumaroyl) afzelin (compound 1). **Figure S16.** HMBC spectrum (400 MHz, CD_3_OD) of 4΄-O-methyl (2″,4″-di-*E*-p-coumaroyl) afzelin (compound 1) (expanded). **Figure S17.** COSY spectrum (400 MHz, CDCl_3_) of 4΄-O-methyl (2″,4″-di-*E*-p-coumaroyl) afzelin (compound 1). **Figure S18.** COSY spectrum (400 MHz, CDCl_3_) of 4΄-O-methyl (2″,4″-di-*E*-p-coumaroyl) afzelin (compound 1) (expanded). **Figure S19.** COSY spectrum (400 MHz, CDCl_3_) of 4΄-O-methyl (2″,4″-di-*E*-p-coumaroyl) afzelin (compound 1) (expanded). **Figure S20.** ESI-MS spectrum of 4΄-O-methyl (2″,4″-di-*E*-p-coumaroyl) afzelin (compound 2). **Figure S21.** ESI-MS spectrum of 4΄-O-methyl (2″,4″-di-*E*-p-coumaroyl) afzelin (compound 2). **Figure S22. **^1^H-NMR spectrum (400 MHz, CD_3_OD) of quercetin 3-O-(2″,4″-di-*E*-p-coumaroyl)-α-L-rhamnopyranoside (compound 2). **Figure S23. **^1^H-NMR spectrum (400 MHz, CD_3_OD) of quercetin 3-O-(2″,4″-di-*E*-p-coumaroyl)-α-L-rhamnopyranoside (compound 2) (expanded). **Figure S24. **^1^H-NMR spectrum (400 MHz, CD_3_OD) of quercetin 3-O-(2″,4″-di-*E*-p-coumaroyl)-α-L-rhamnopyranoside (compound 2) (expanded). **Figure S25.** DEPT-135 spectrum (100 MHz, CD_3_OD) of 4΄-O-methyl (2″,4″-di-*E*-p-coumaroyl) afzelin (compound 2). **Figure S26.** DEPT-135 spectrum (100 MHz, CD_3_OD) of 4΄-O-methyl (2″,4″-di-*E*-p-coumaroyl) afzelin (compound 2). **Figure S27.** HSQC spectrum (400 MHz, CD_3_OD) of 4΄-O-methyl (2″,4″-di-*E*-p-coumaroyl) afzelin (compound 2). **Figure S28.** HMBC spectrum (400 MHz, CD_3_OD) of 4΄-O-methyl (2″,4″-di-*E*-p-coumaroyl) afzelin (compound 2). **Figure S29.** COSY spectrum (400 MHz, CD_3_OD) of 4΄-O-methyl (2″,4″-di-*E*-p-coumaroyl) afzelin (compound 2). **Figure S30.** COSY spectrum (400 MHz, CD_3_OD) of 4΄-O-methyl (2″,4″-di-*E*-p-coumaroyl) afzelin (compound 2) (expanded). **Figure S31.** COSY spectrum (400 MHz, CD_3_OD) of 4΄-O-methyl (2″,4″-di-*E*-p-coumaroyl) afzelin (compound 2) (expanded). **Figure S32.** The collected Swiss Albino Mice for conducting pharmacological studies of *L. glutinosa*.

## Data Availability

The manuscript and supplementary file contain all the necessary data that support the findings and conclusion of the research.

## Abbreviations

ADMET: Absorption, Distribution, Metabolism, Excretion, and Toxicity
DM: Diabetes Mellitus
GPC: Gel Permeation Chromatography
LGC: Chloroform soluble fraction of *Litsea glutinosa*
MELG: Methanol Extract of *L. glutinosa*
NMR: Nuclear Magnetic Resonance
PASS: Prediction of Activity Spectra for Substances
PDB: Protein Data Bank
STZ: Streptozotocin
TLC: Thin Layer Chromatography
TMS: Tetramethyl Silane
WHO: World Health Organization
