# Supplementary material for: Two rare flavonoid glycosides from Litsea glutinosa (Lour.) C. B. Rob.: experimental and computational approaches endorse antidiabetic potentiality
Source: BMC Complement Med Ther. 2024 Feb 1;24:69. doi: 10.1186/s12906-024-04337-0 (PMC10832099; doi:10.1186/s12906-024-04337-0)
Supplement: Supplementary file 1 — Additional file 1: Figure S1. ESI-MS spectrum of 4΄-O-methyl (2″,4″-di-E-p-coumaroyl) afzelin (compound 1). Figure S2. ESI-MS spectrum of 4΄-O-methyl (2″,4″-di-E-p-coumaroyl) afzelin (compound 1). Figure S3. 1H-NMR spectrum (400 MHz, CD3OD) of 4΄-O-methyl (2″,4″-di-E-p-coumaroyl) afzelin (compound 1). Figure S4. 1H-NMR spectrum (400 MHz, CD3OD) of 4΄-O-methyl (2″,4″-di-E-p-coumaroyl) afzelin (compound 1) (expanded). Figure S5. 1H-NMR spectrum (400 MHz, CD3OD) of 4΄-O-methyl (2″,4″-di-E-p-coumaroyl) afzelin (compound 1) (expanded). Figure S6. 1H-NMR spectrum (400 MHz, CD3OD) of 4΄-O-methyl (2″,4″-di-E-p-coumaroyl) afzelin (compound 1) (expanded). Figure S7. 1H-NMR spectrum (400 MHz, CD3OD) of 4΄-O-methyl (2″,4″-di-E-p-coumaroyl) afzelin (compound 1) (expanded). Figure S8. 13C-NMR spectrum (100 MHz, CD3OD) of 4΄-O-methyl (2″,4″-di-E-p-coumaroyl) afzelin (compound 1). Figure S9. 13C-NMR spectrum (100 MHz, CD3OD) of 4΄-O-methyl (2″,4″-di-E-p-coumaroyl) afzelin (compound 1) (expanded). Figure S10. 13C-NMR spectrum (100 MHz, CD3OD) of 4΄-O-methyl (2″,4″-di-E-p-coumaroyl) afzelin (compound 1) (expanded). Figure S11. 13C-NMR spectrum (100 MHz, CD3OD) of 4΄-O-methyl (2″,4″-di-E-p-coumaroyl) afzelin (compound 1) (expanded). Figure S12. HSQC spectrum (400 MHz, CD3OD) of 4΄-O-methyl (2″,4″-di-E-p-coumaroyl) afzelin (compound 1). Figure S13. HSQC spectrum (400 MHz, CD3OD) of 4΄-O-methyl (2″,4″-di-E-p-coumaroyl) afzelin (compound 1) (expanded). Figure S14. HSQC spectrum (400 MHz, CD3OD) of 4΄-O-methyl (2″,4″-di-E-p-coumaroyl) afzelin (compound 1) (expanded). Figure S15. HMBC spectrum (400 MHz, CD3OD) of 4΄-O-methyl (2″,4″-di-E-p-coumaroyl) afzelin (compound 1). Figure S16. HMBC spectrum (400 MHz, CD3OD) of 4΄-O-methyl (2″,4″-di-E-p-coumaroyl) afzelin (compound 1) (expanded). Figure S17. COSY spectrum (400 MHz, CDCl3) of 4΄-O-methyl (2″,4″-di-E-p-coumaroyl) afzelin (compound 1). Figure S18. COSY spectrum (400 MHz, CDCl3) of 4΄-O-methyl (2″,4″-di-E-p-coumaroyl) afzelin (compound 1) [file 12906_2024_4337_MOESM1_ESM.pdf]

Supplementary file

Two Rare Flavonoid Glycosides from *Litsea glutinosa* (Lour.) C. B. Rob.: Experimental and Computational Approaches Endorse Antidiabetic Potentiality

Short Title: *Flavonoid Glycosides from Litsea glutinosa (Lour.) C. B. Rob.*

Israt Jahan Bulbul[1], Md. Jamal Hossain[2*], Mohammad Rashedul Haque[3], Muhammad Abdullah Al-Mansur[4], Choudhury M. Hasan[3], Abdullah Al Hasan[1], and Mohammad A. Rashid[3*]

[1] *Department of Pharmacy, Southeast University, Banani, Dhaka-1213, Bangladesh*

[2] *Department of Pharmacy, School of Pharmaceutical Sciences, State University of Bangladesh, 77 Satmasjid Road, Dhanmondi, Dhaka 1205, Bangladesh*

[3] *Phytochemical Research Laboratory, Department of Pharmaceutical Chemistry, Faculty of Pharmacy, University of Dhaka, Dhaka-1000, Bangladesh*

[4] *Bangladesh Council of Scientific and Industrial Research (BCSIR), Dr. Qudrat-I-Khuda Road, Dhanmondi, Dhaka-1205, Bangladesh*

***Corresponding author:**

Md. Jamal Hossain

Assistant Professor, Department of Pharmacy, School of Pharmaceutical Sciences, State University of Bangladesh, 77 Satmasjid Road, Dhanmondi, Dhaka 1205, Bangladesh

E-mail: jamal.du.p48@gmail.com; jamalhossain@sub.edu.bd

Prof. Dr. Mohammad A. Rashid

Phytochemical Research Laboratory, Department of Pharmaceutical Chemistry, Faculty of Pharmacy, University of Dhaka, Dhaka-1000, Bangladesh

E-mail: arpharm64@du.ac.bd

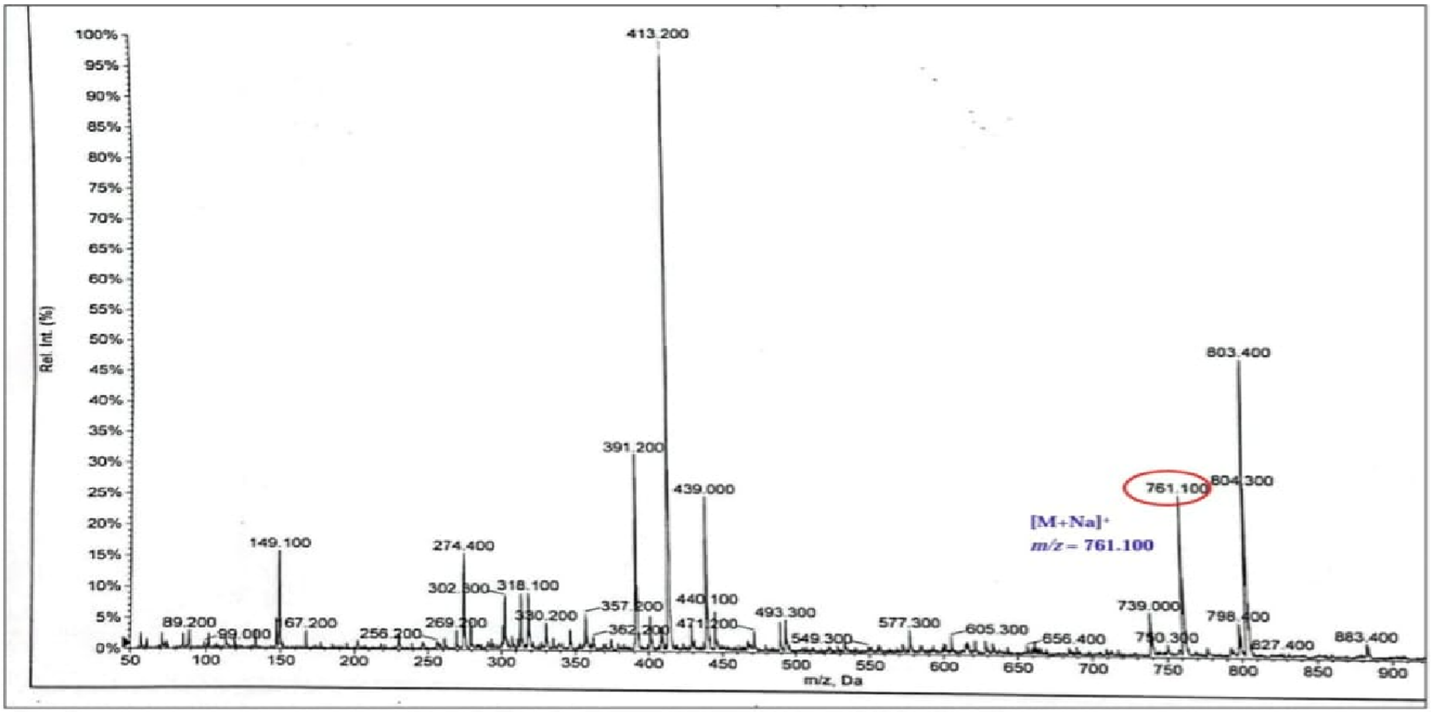

Figure S1. ESI-MS spectrum of 4′-O-methyl (2″,4″-di-E-p-coumaroyl) afzelin (compound **1**)

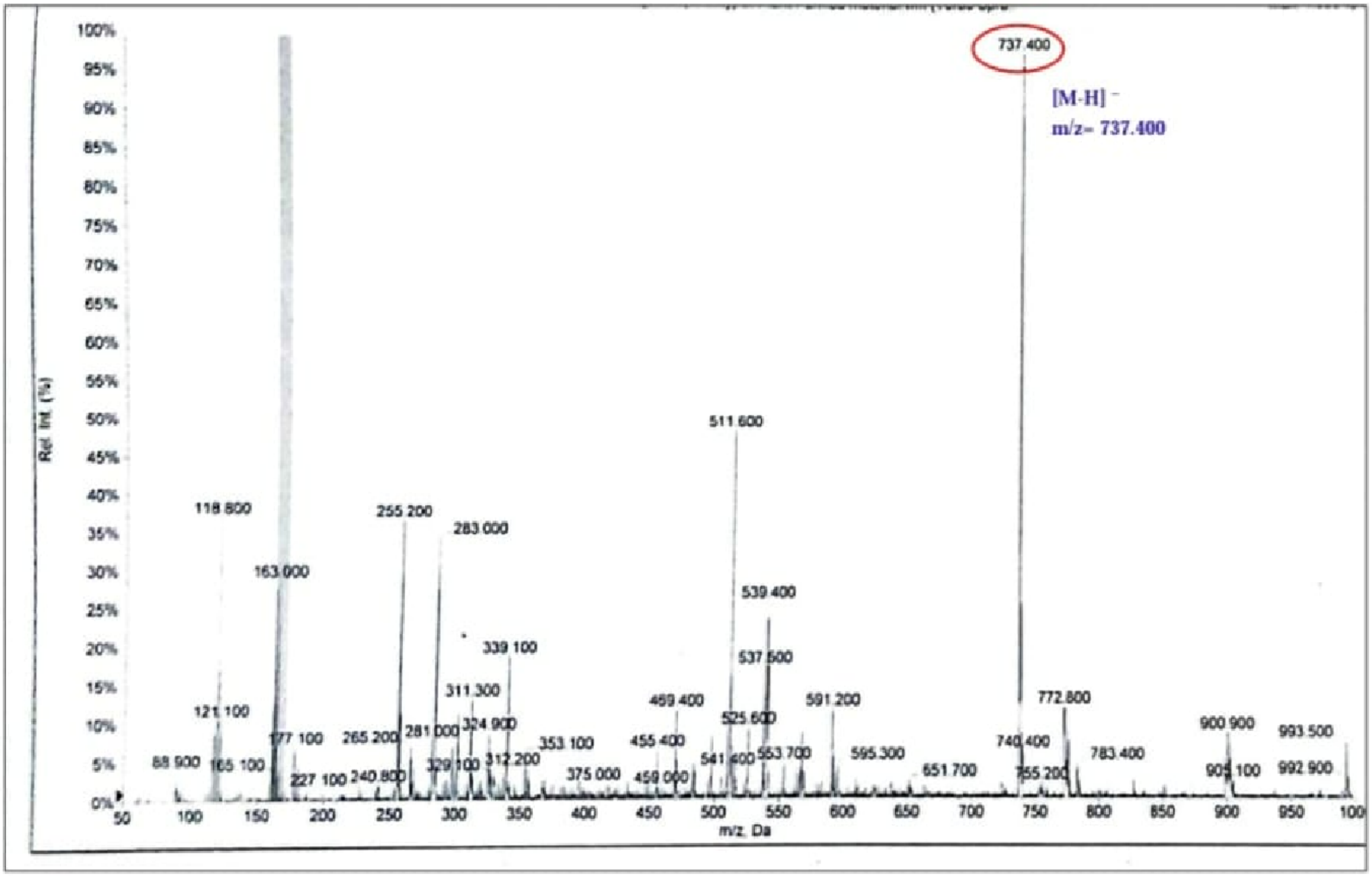

Figure S2. ESI-MS spectrum of 4′-O-methyl (2″,4″-di-E-p-coumaroyl) afzelin (compound **1**)

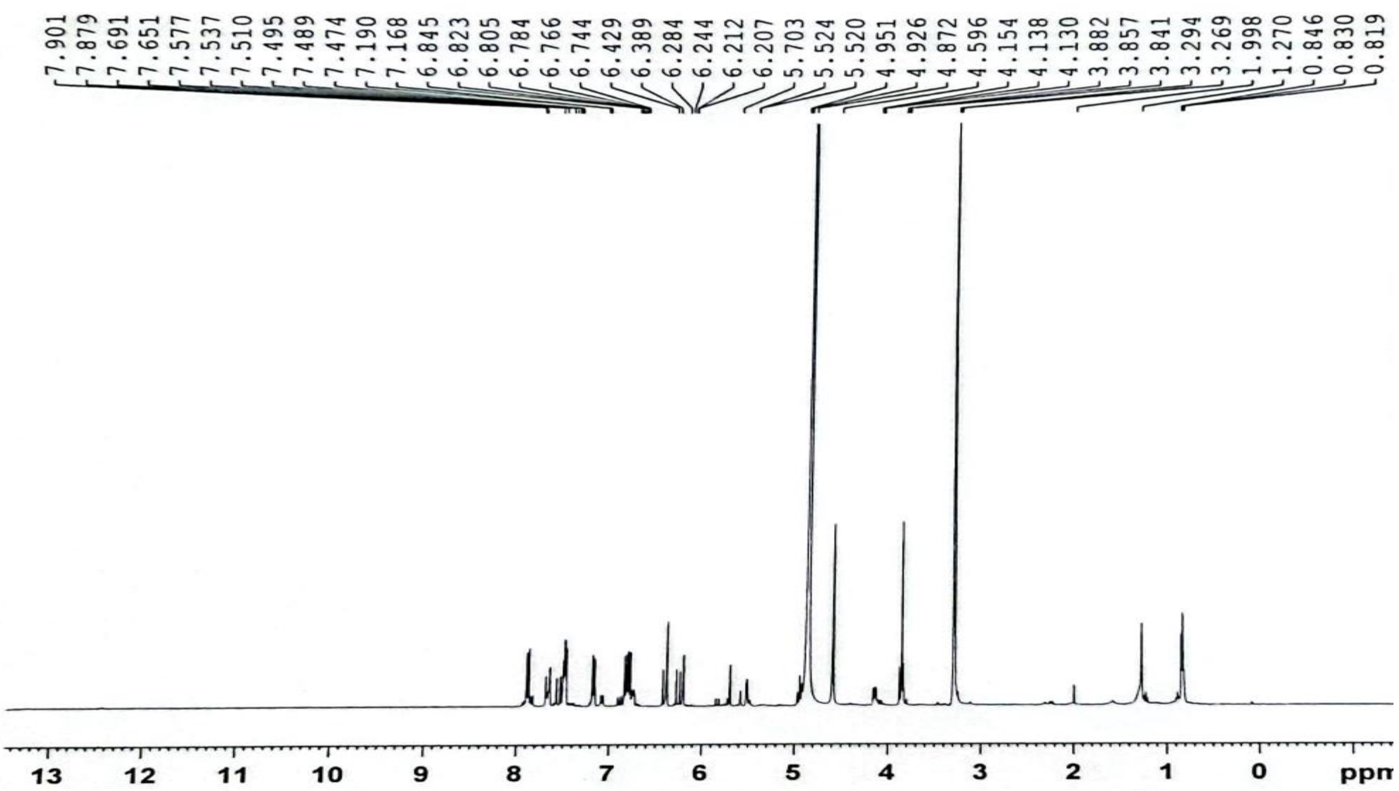

Figure S3. ^{1}H-NMR spectrum (400 MHz, CD_3OD) of 4′-O-methyl (2″,4″-di-E-p-coumaroyl) afzelin (compound **1**).

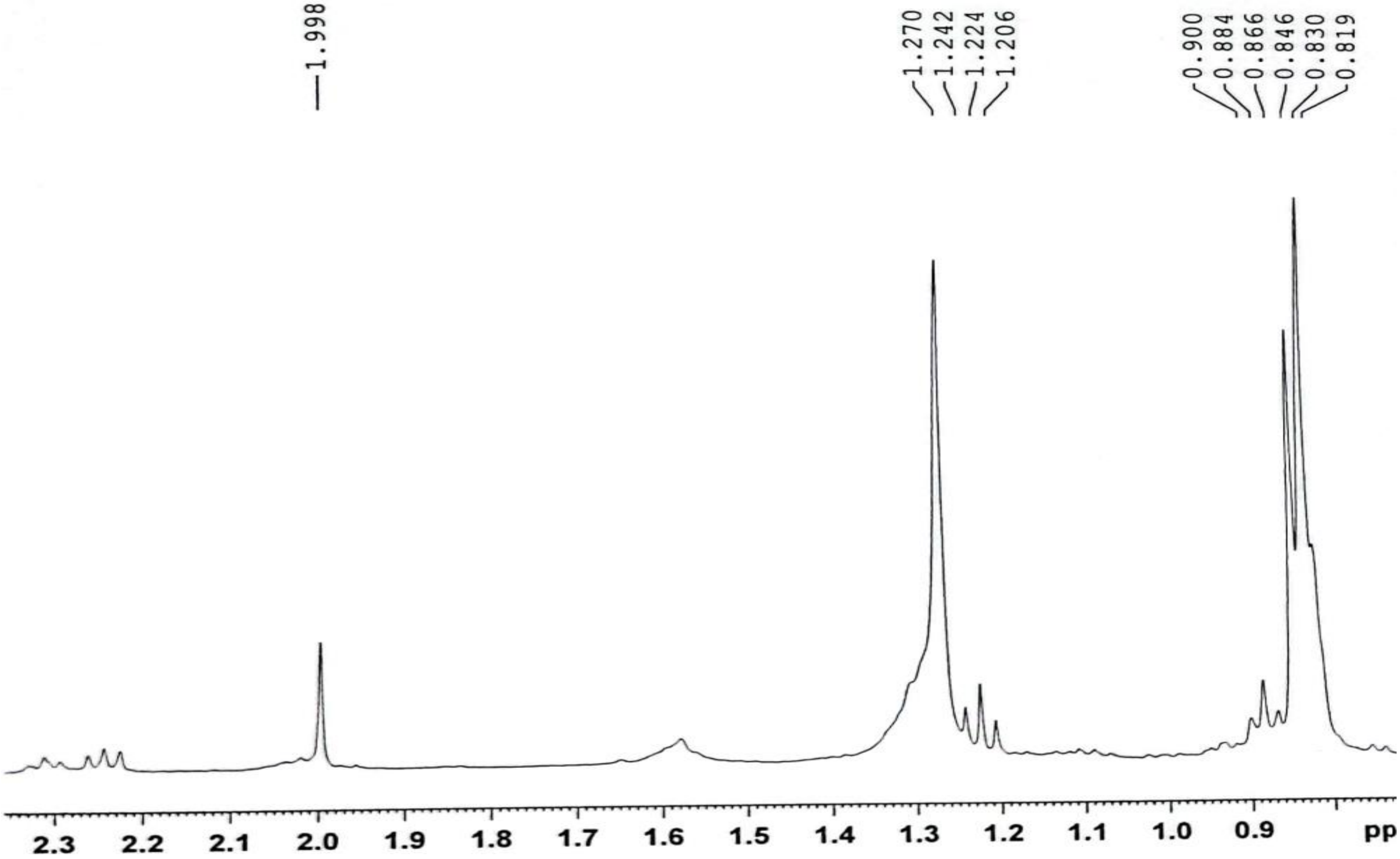

Figure S4. ^{1}H-NMR spectrum (400 MHz, CD_3OD) of 4′-O-methyl (2″,4″-di-E-p-coumaroyl) afzelin (compound **1**) (expanded).

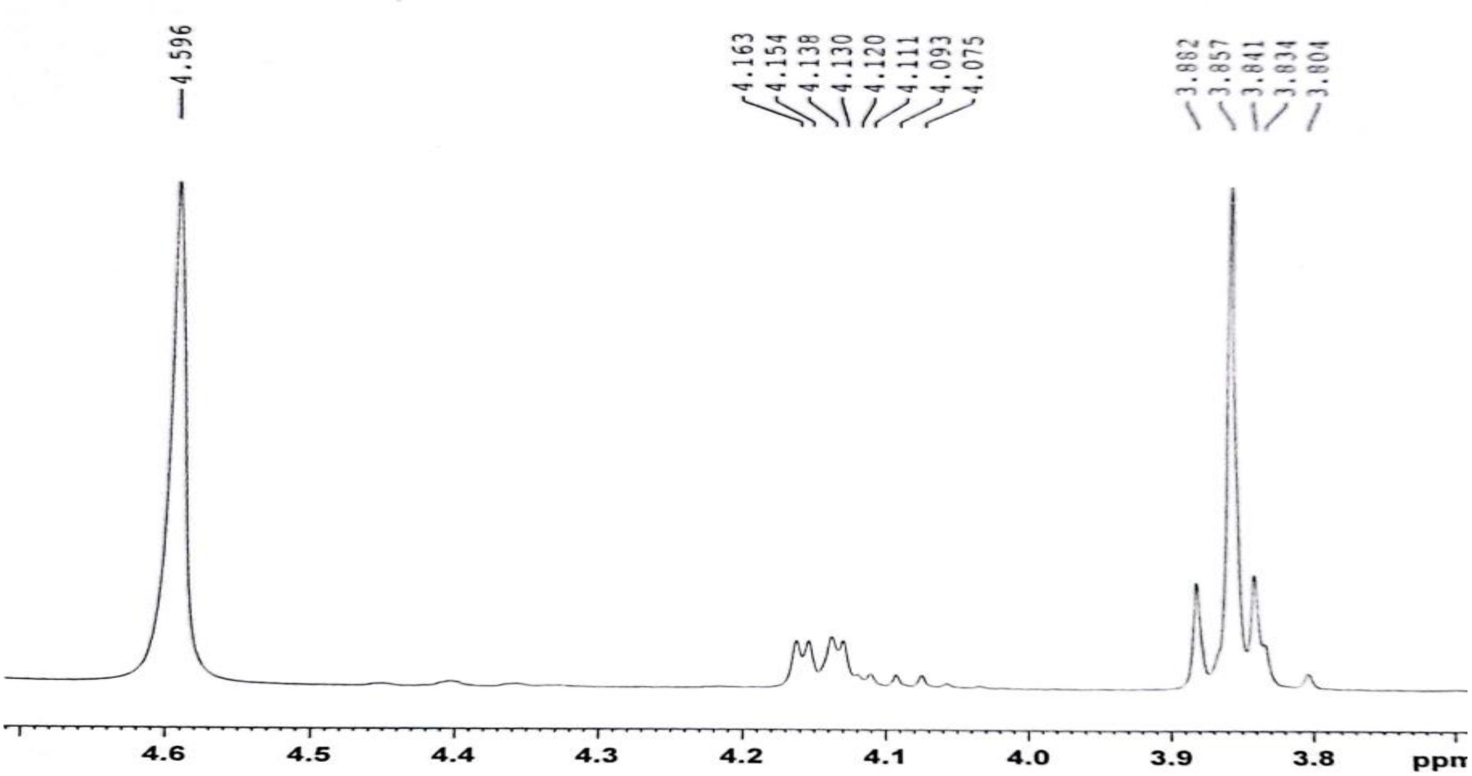

Figure S5. ^{1}H-NMR spectrum (400 MHz, CD_3OD) of 4′-O-methyl (2″,4″-di-E-p-coumaroyl) afzelin (compound **1**) (expanded).

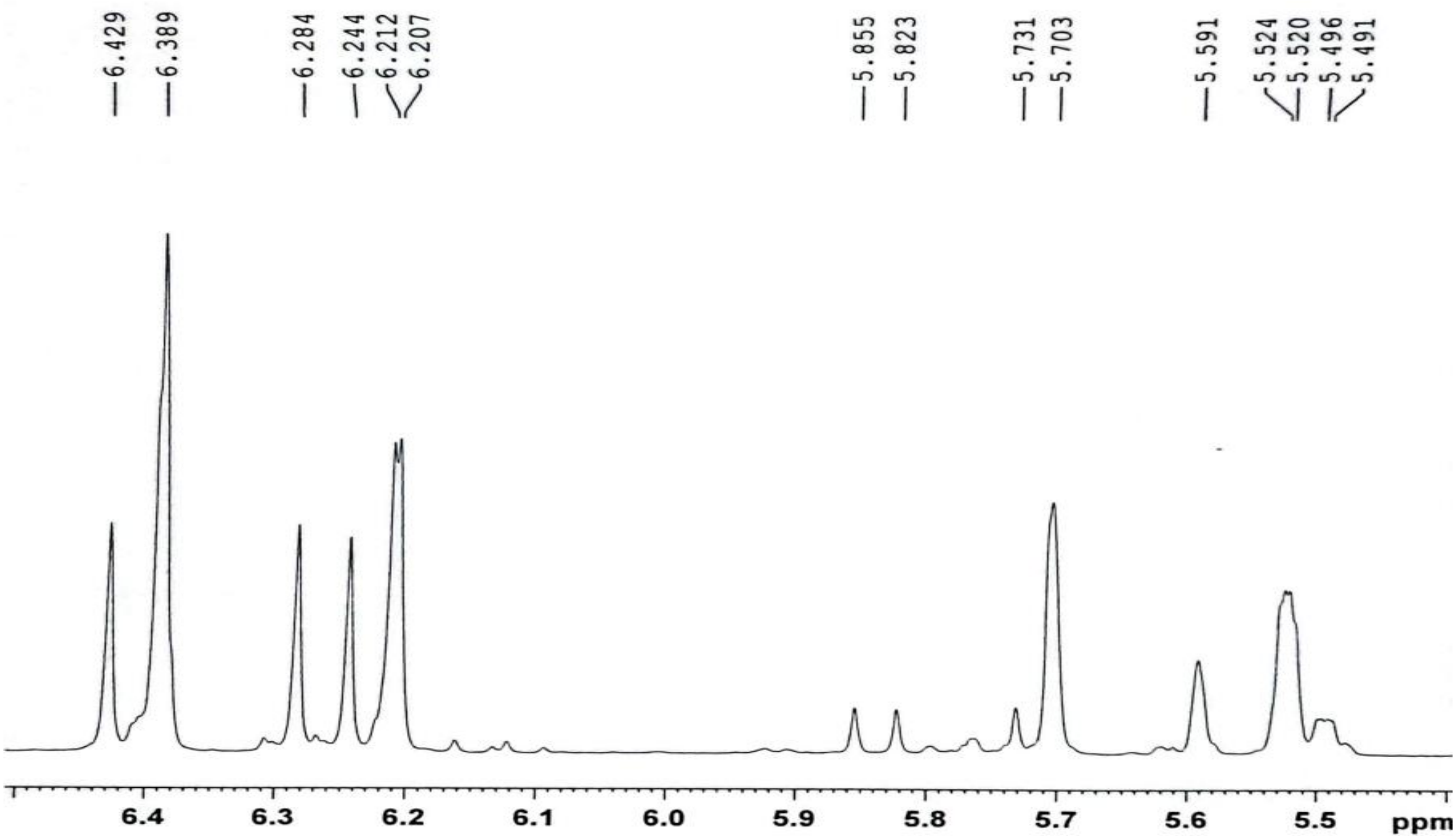

Figure S6. ^{1}H-NMR spectrum (400 MHz, CD_3OD) of 4′-O-methyl (2″,4″-di-E-p-coumaroyl) afzelin (compound **1**) (expanded).

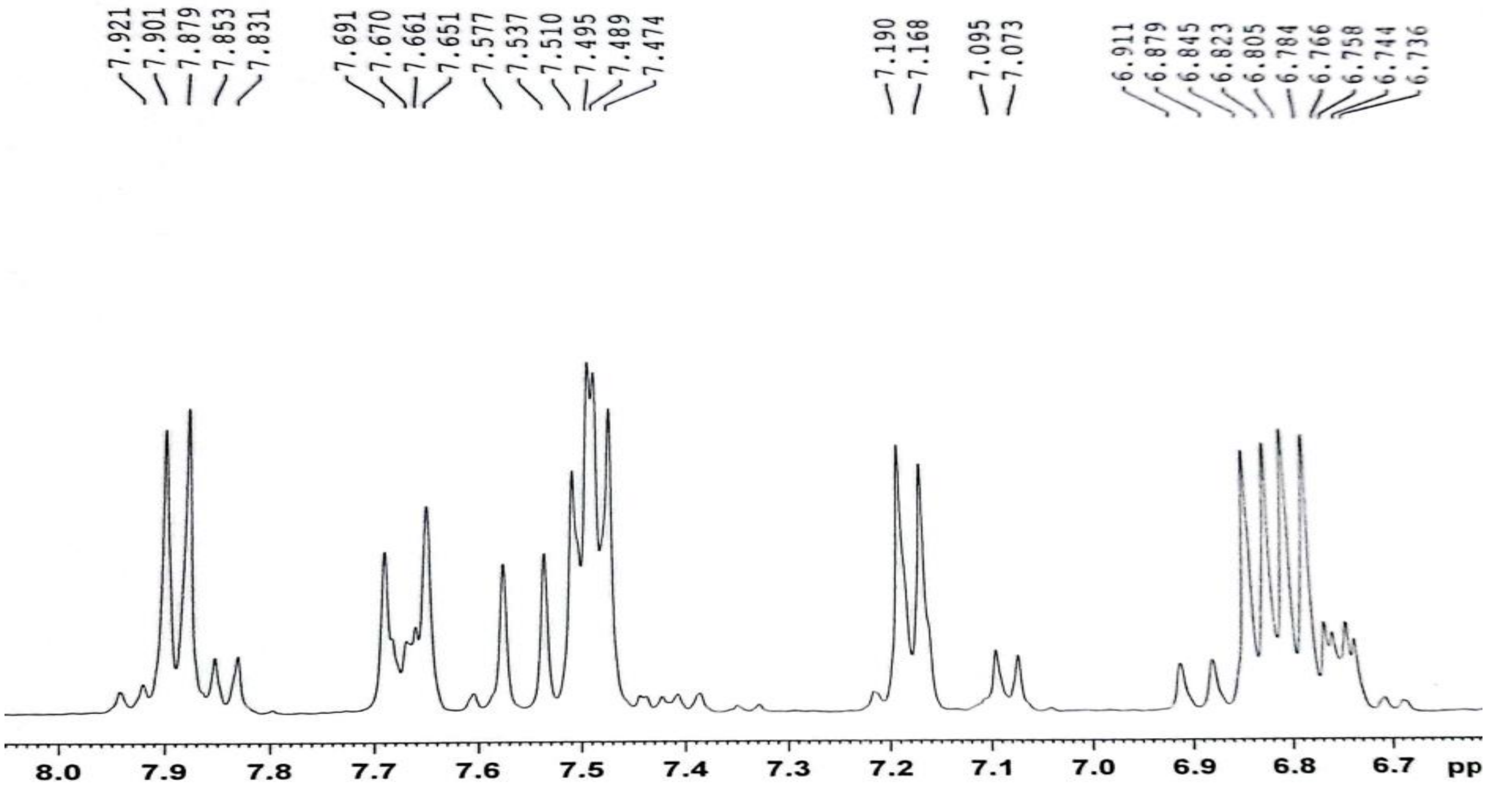

Figure S7. ^{1}H-NMR spectrum (400 MHz, CD_3OD) of 4´-O-methyl (2″,4″-di-E-p-coumaroyl) afzelin (compound **1**) (expanded).

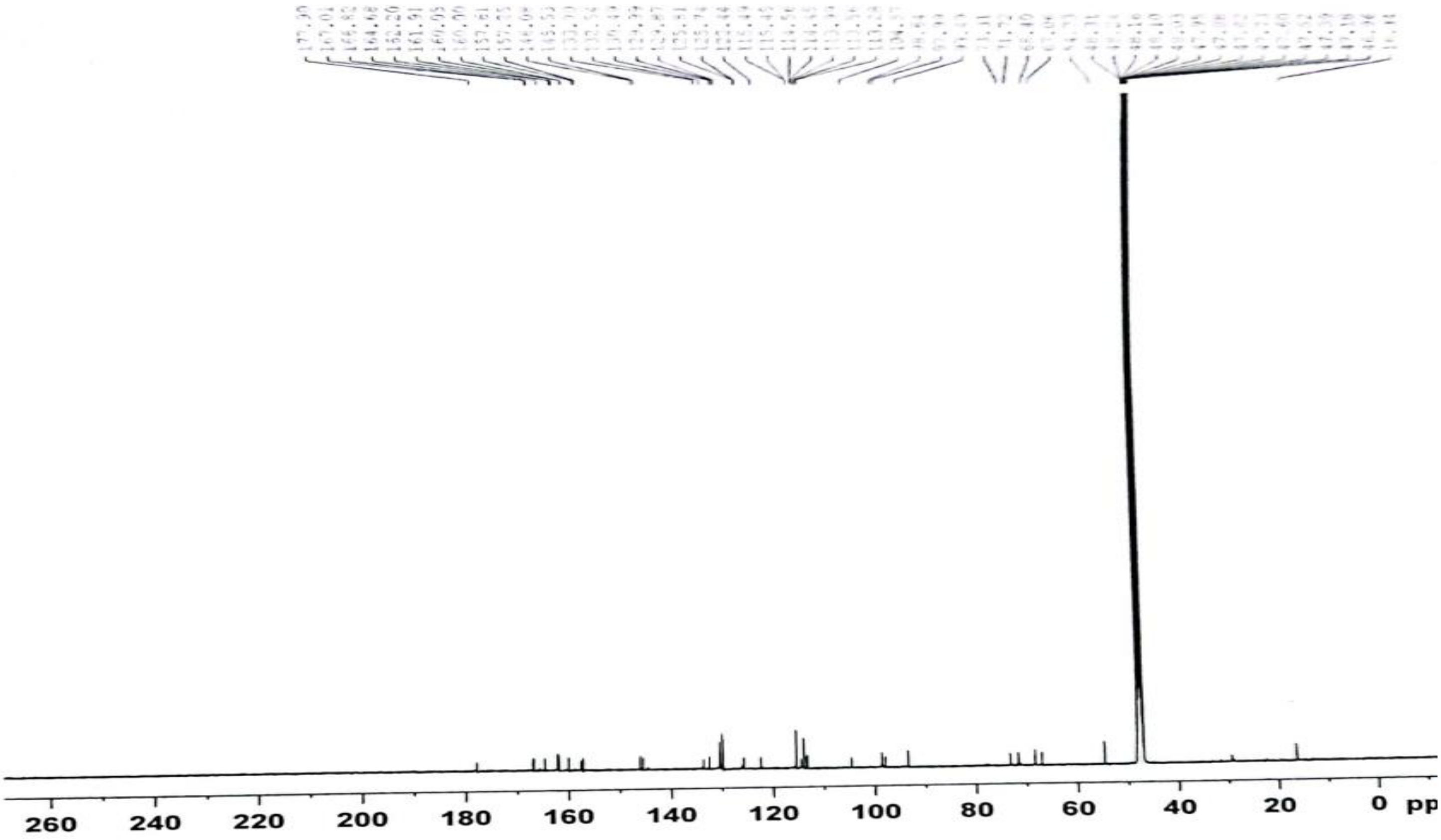

Figure S8. [13] C-NMR spectrum (100 MHz, CD_3OD) of 4´-O-methyl (2″,4″-di-E-p-coumaroyl) afzelin (compound **1**).

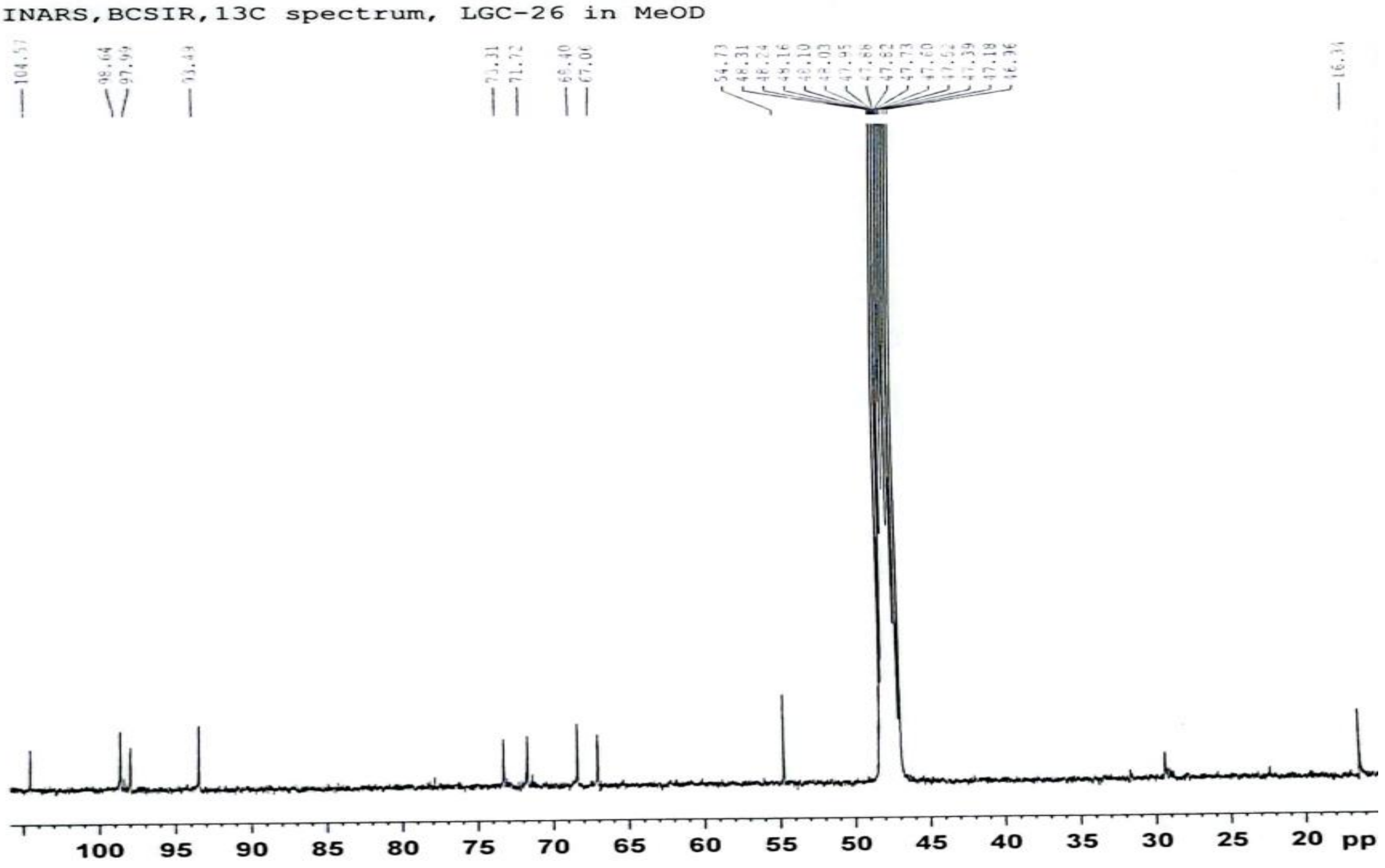

Figure S9. ^{13}C-NMR spectrum (100 MHz, CD_3OD) of 4′-O-methyl (2″,4″-di-E-p-coumaroyl) afzelin (compound **1**) (expanded).

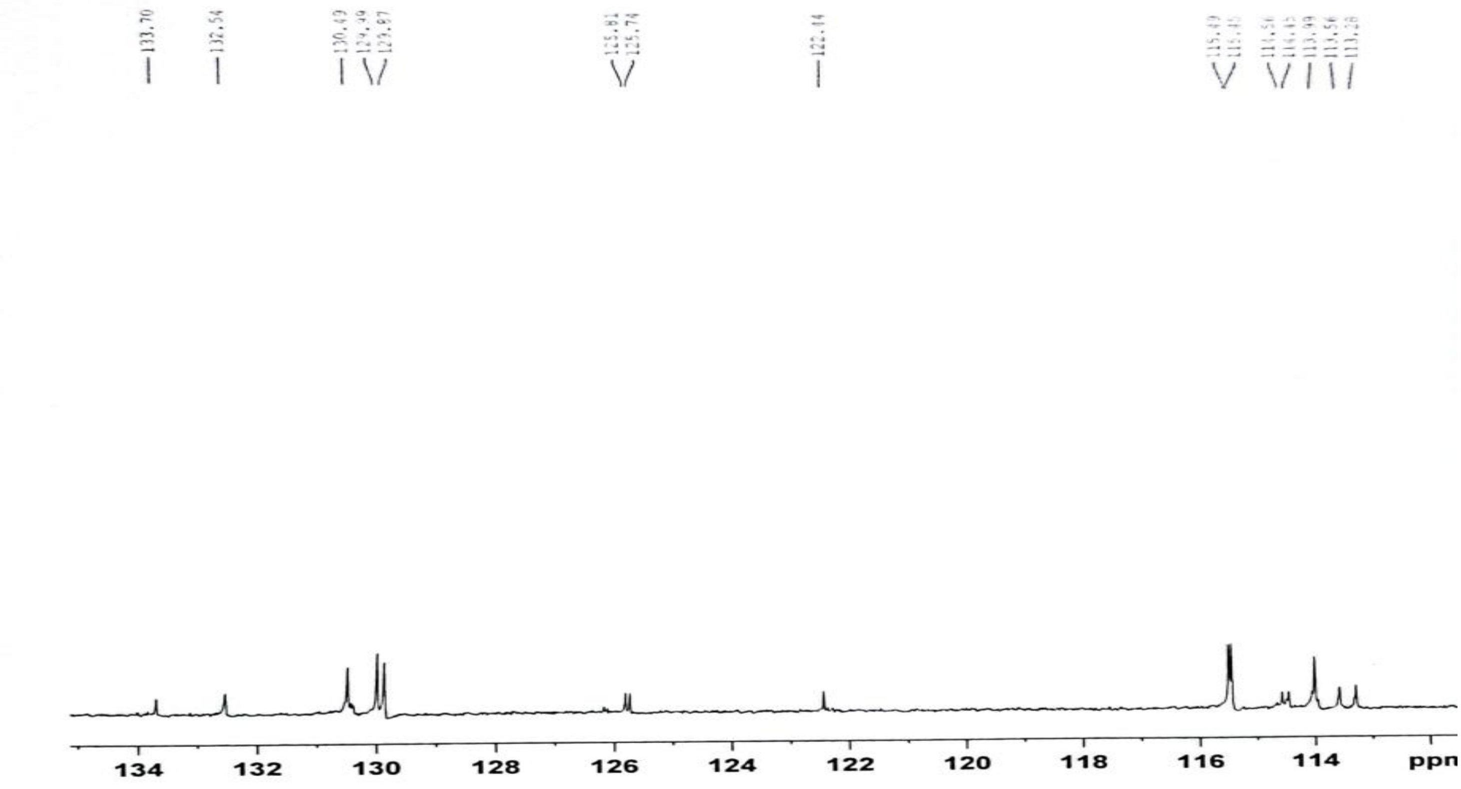

Figure S10. ^{13}C-NMR spectrum (100 MHz, CD_3OD) of 4′-O-methyl (2″,4″-di-E-p-coumaroyl) afzelin (compound **1**) (expanded).

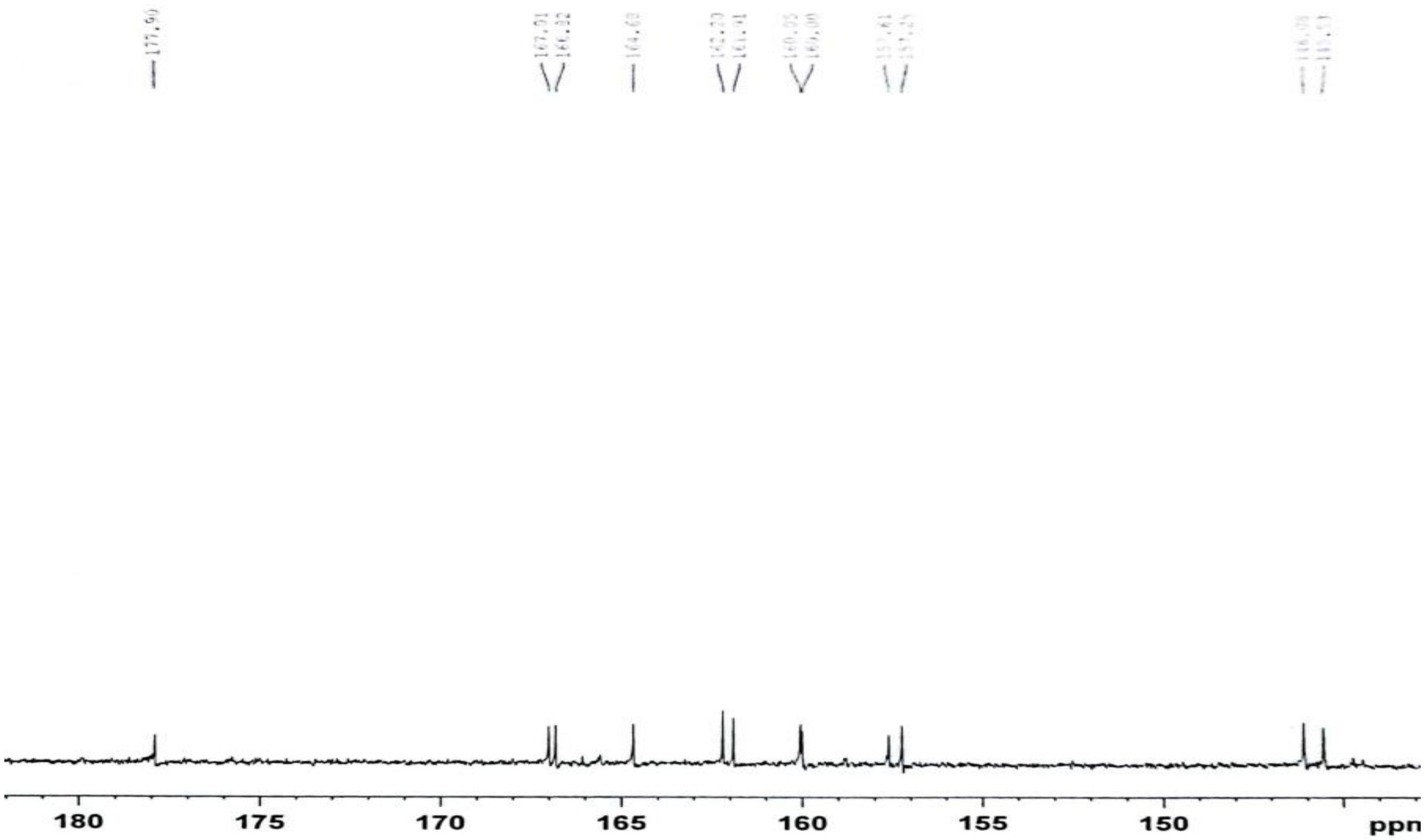

Figure S11. [13] C-NMR spectrum (100 MHz, CD_3OD) of 4′-O-methyl (2″,4″-di-E-p-coumaroyl) afzelin (compound **1**) (expanded).

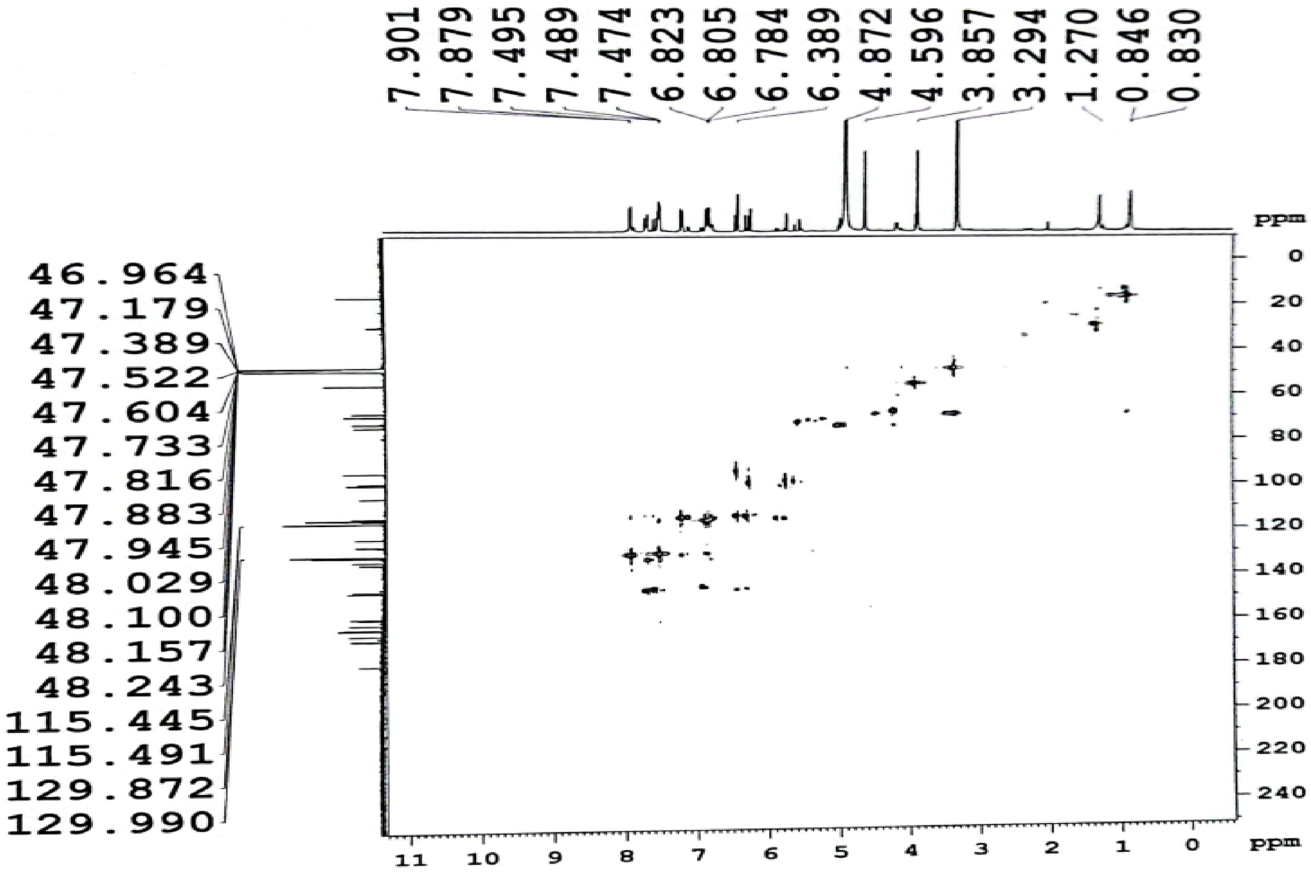

Figure S12. HSQC spectrum (400 MHz, CD_3OD) of 4′-O-methyl (2″,4″-di-E-p-coumaroyl) afzelin (compound **1**).

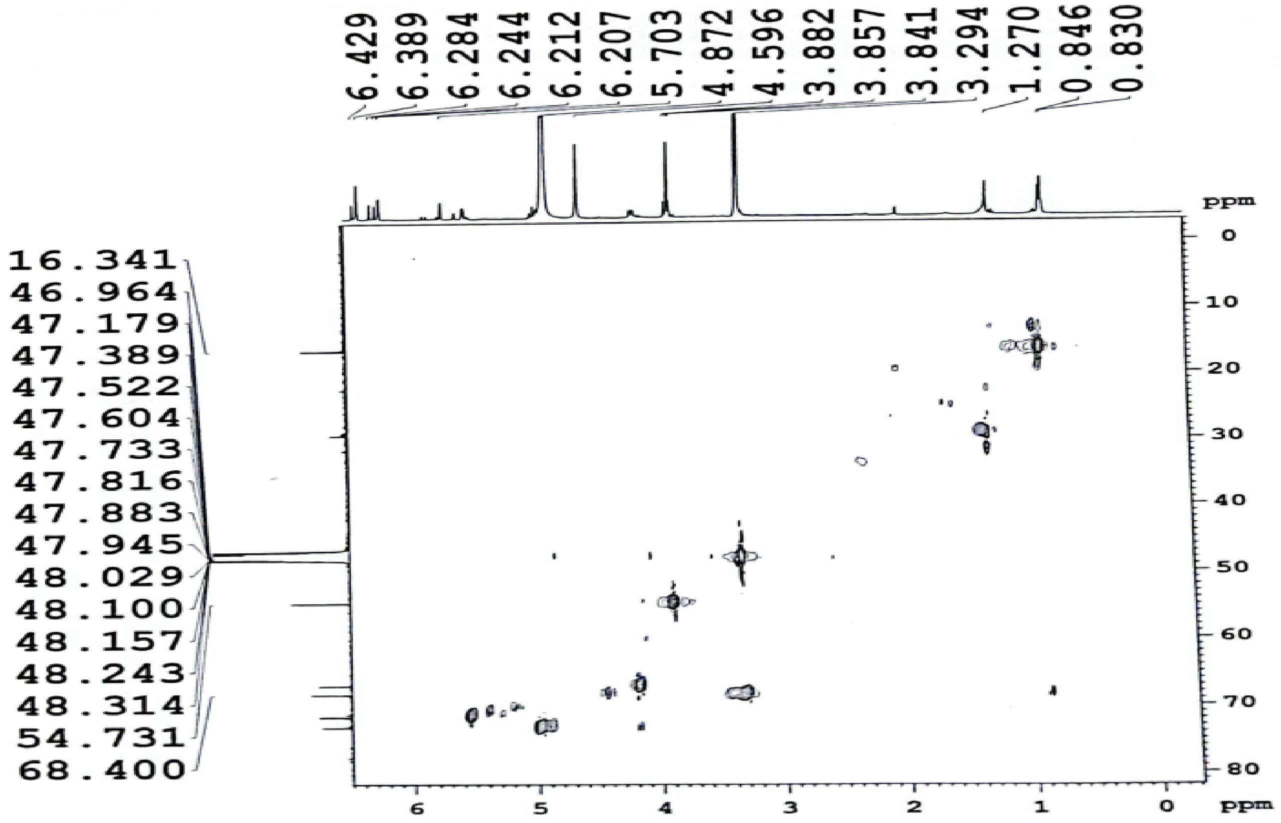

Figure S13. HSQC spectrum (400 MHz, CD_3OD) of 4′-O-methyl (2″,4″-di-E-p-coumaroyl) afzelin (compound **1**) (expanded).

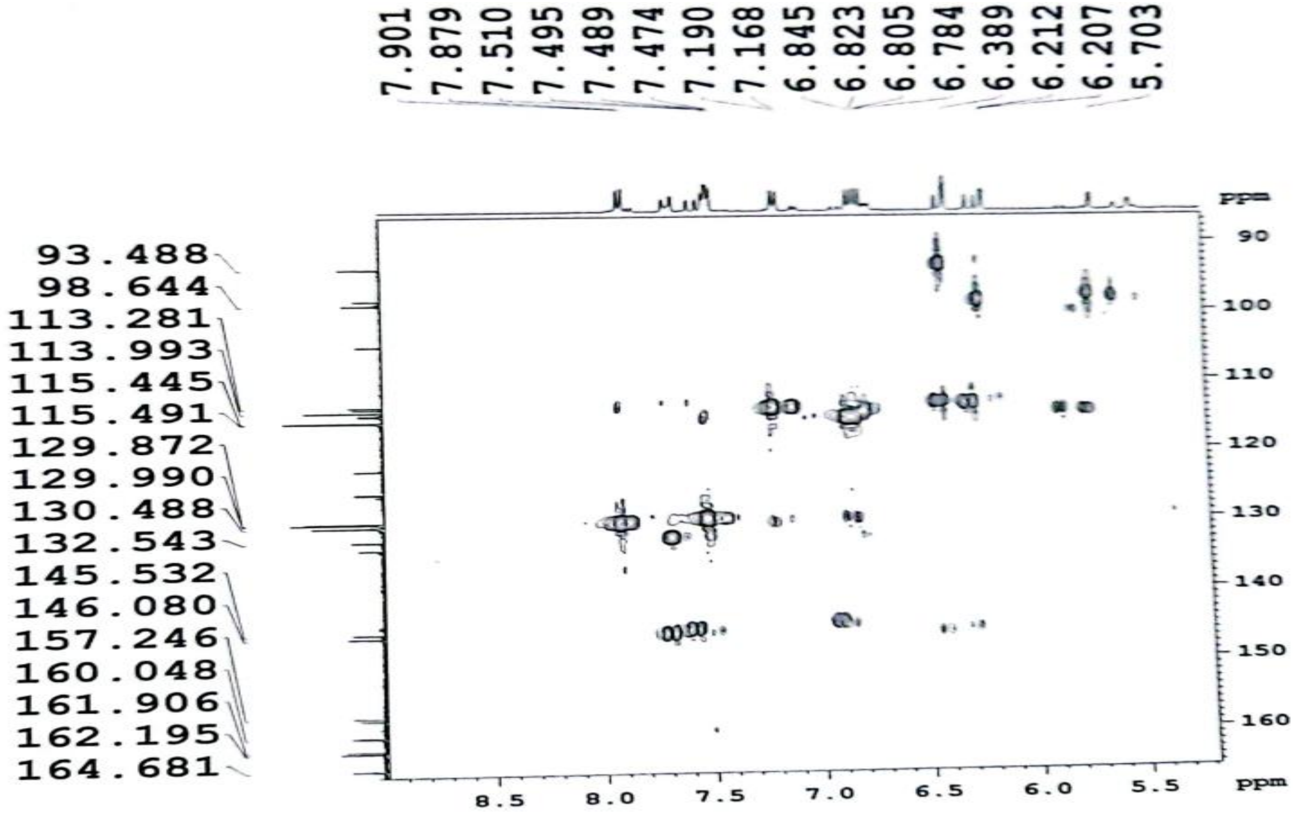

Figure S14. HSQC spectrum (400 MHz, CD_3OD) of 4′-O-methyl (2″,4″-di-E-p-coumaroyl) afzelin (compound **1**) (expanded).

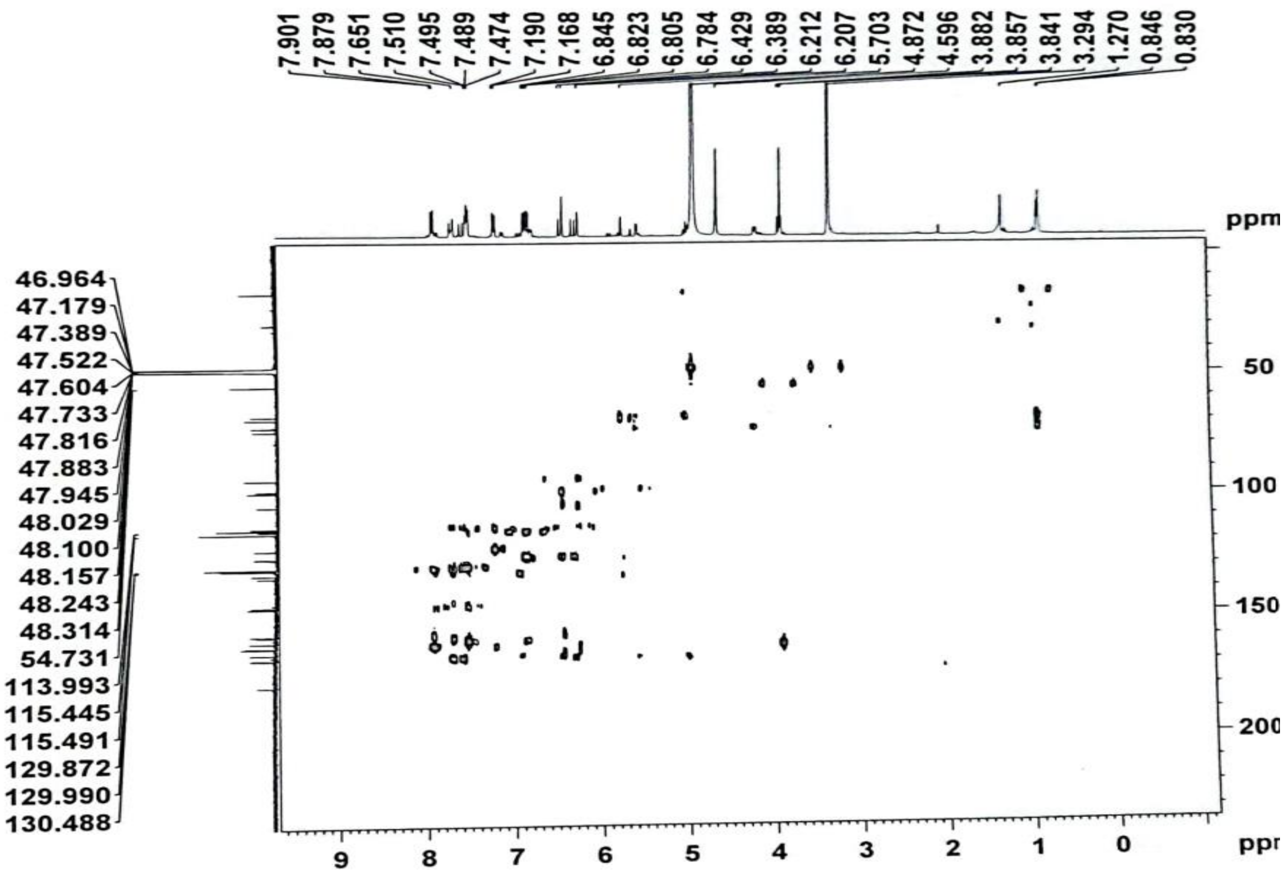

Figure S15. HMBC spectrum (400 MHz, CD_3OD) of 4′-O-methyl (2″,4″-di-E-p-coumaroyl) afzelin (compound **1**).

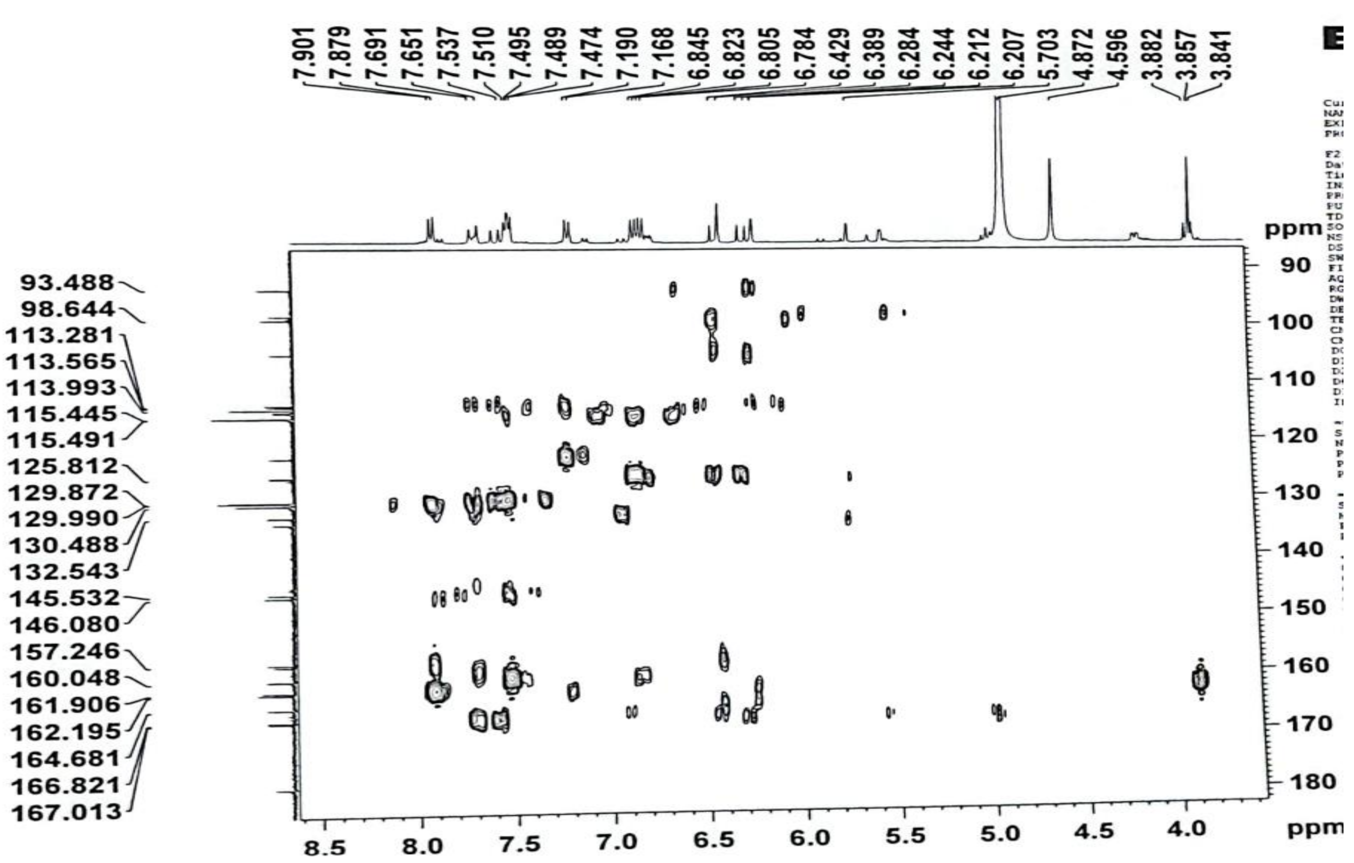

Figure S16. HMBC spectrum (400 MHz, CD_3OD) of 4′-O-methyl (2″,4″-di-E-p-coumaroyl) afzelin (compound **1**) (expanded).

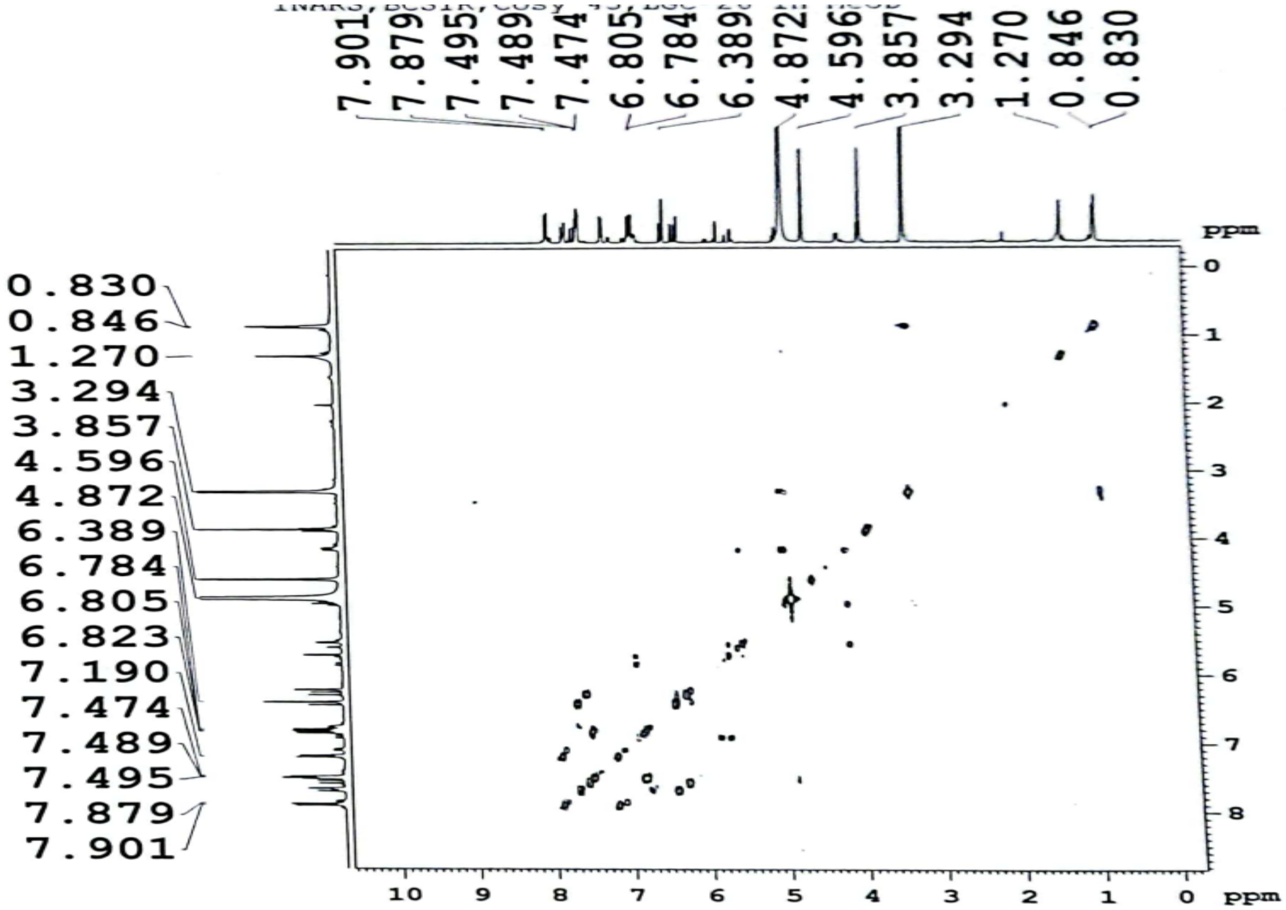

Figure S17: COSY spectrum (400 MHz, $CDCl_3$) of 4′-O-methyl (2″,4″-di-E-p-coumaroyl) afzelin (compound **1**)

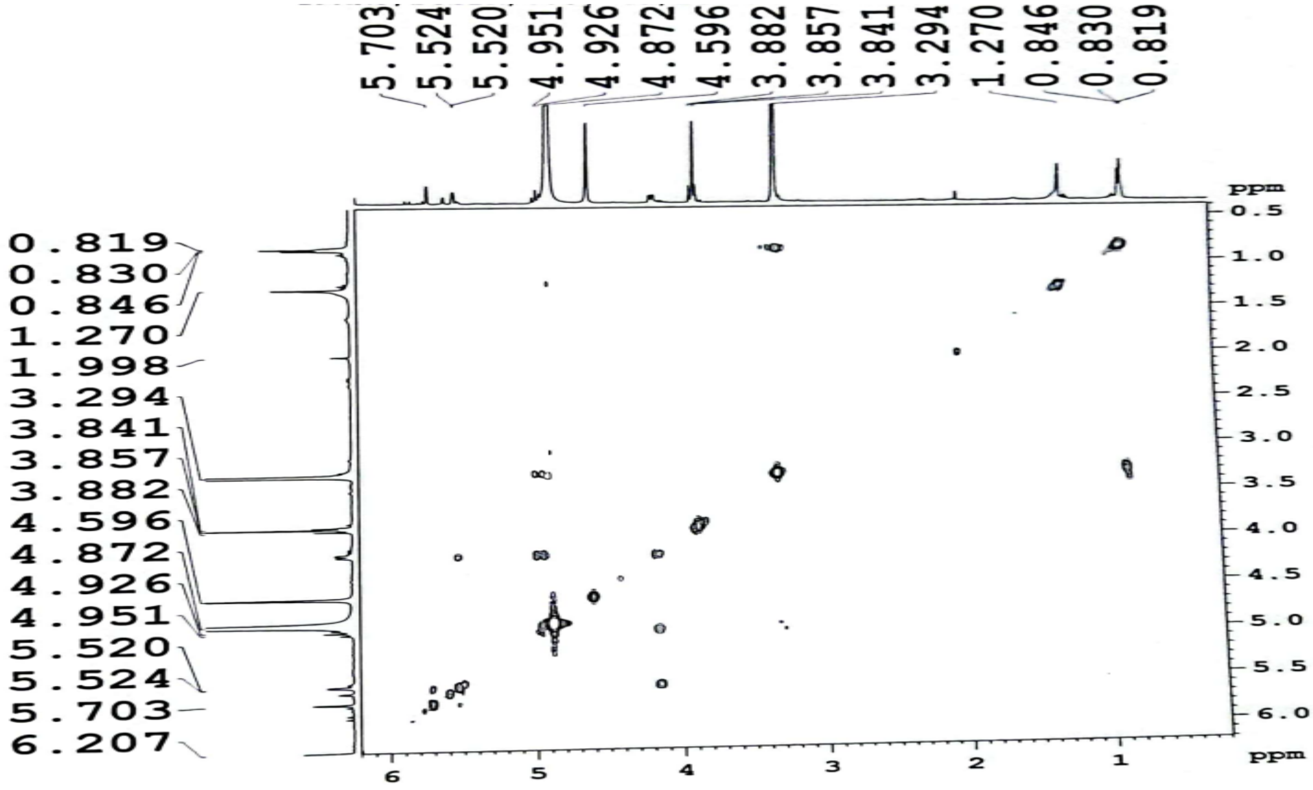

Figure S18: COSY spectrum (400 MHz, $CDCl_3$) of 4′-O-methyl (2″,4″-di-E-p-coumaroyl) afzelin (compound **1**) (expanded).

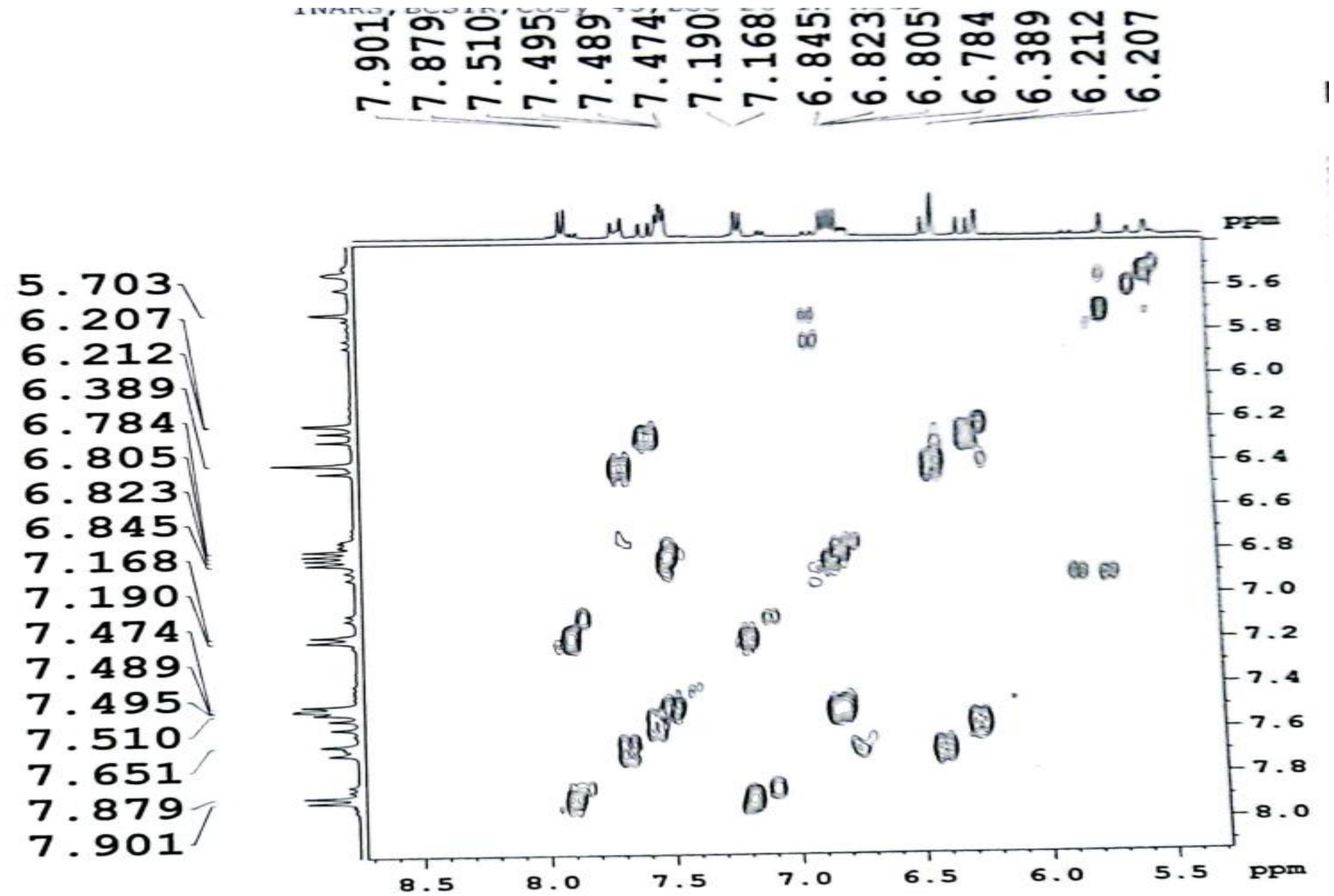

Figure S19: COSY spectrum (400 MHz, $CDCl_3$) of 4′-O-methyl (2″,4″-di-E-p-coumaroyl) afzelin (compound **1**) (expanded).

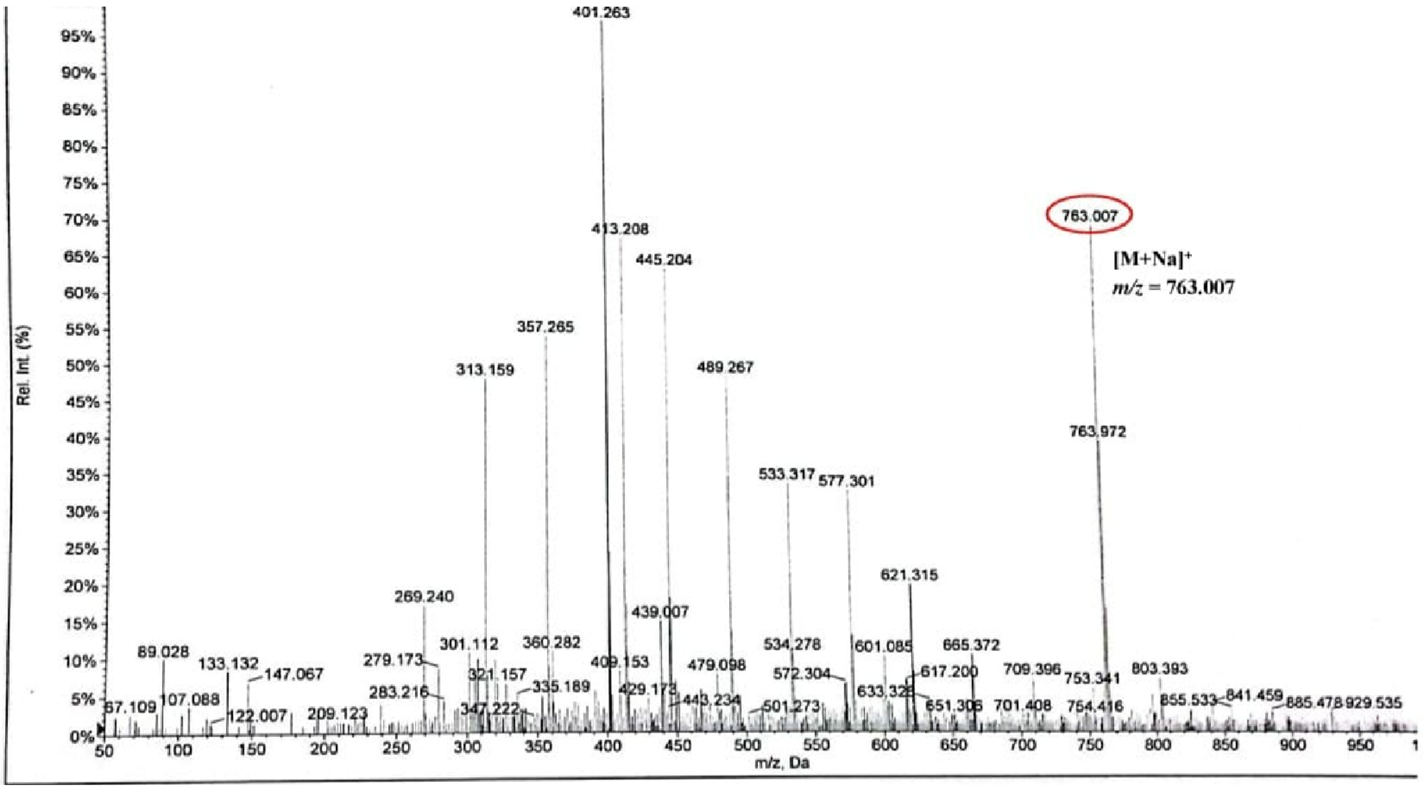

Figure S20. ESI-MS spectrum of 4´-O-methyl (2˝,4˝-di-E-p-coumaroyl) afzelin (compound **2**).

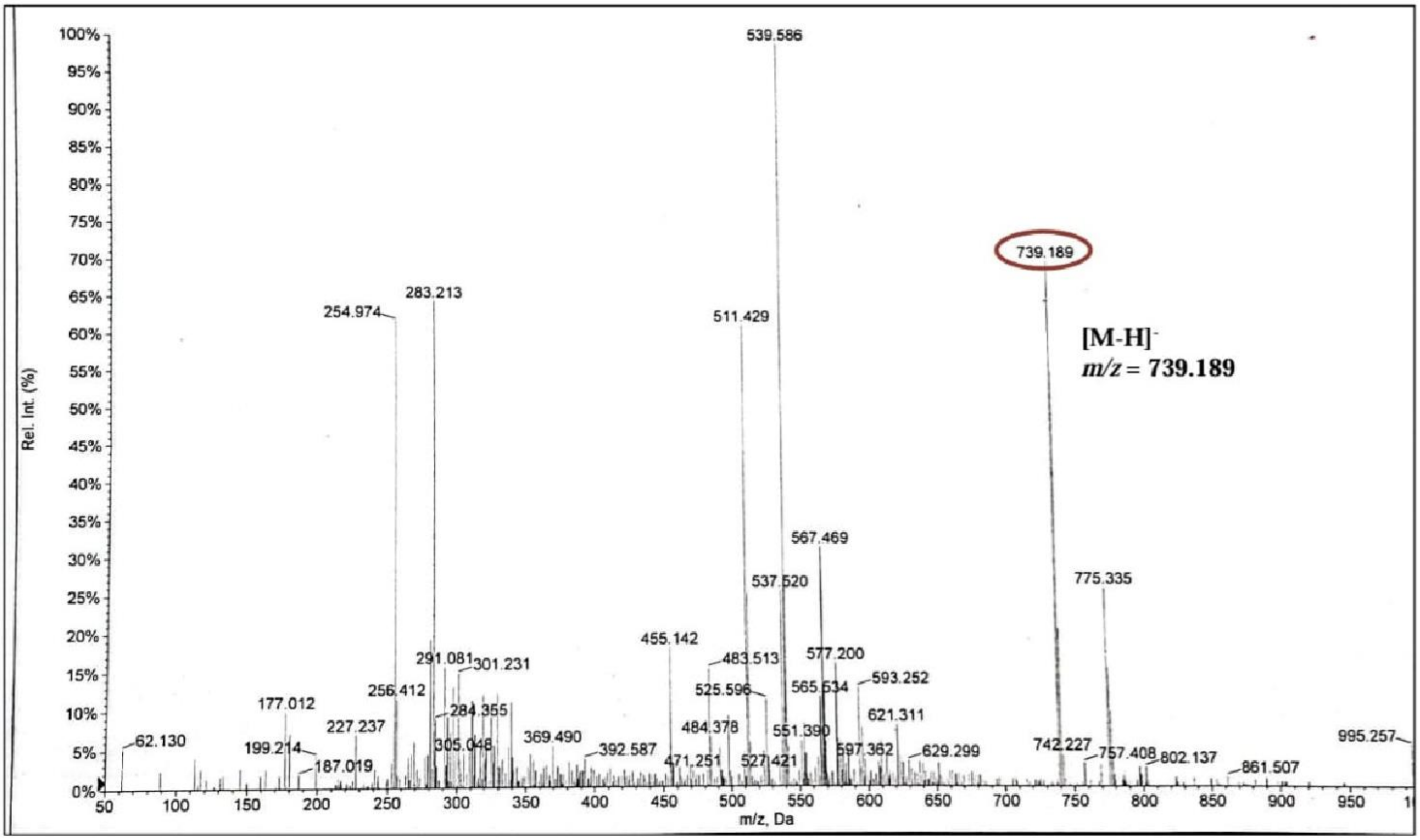

Figure S21. ESI-MS spectrum of 4´-O-methyl (2˝,4˝-di-E-p-coumaroyl) afzelin (compound **2**).

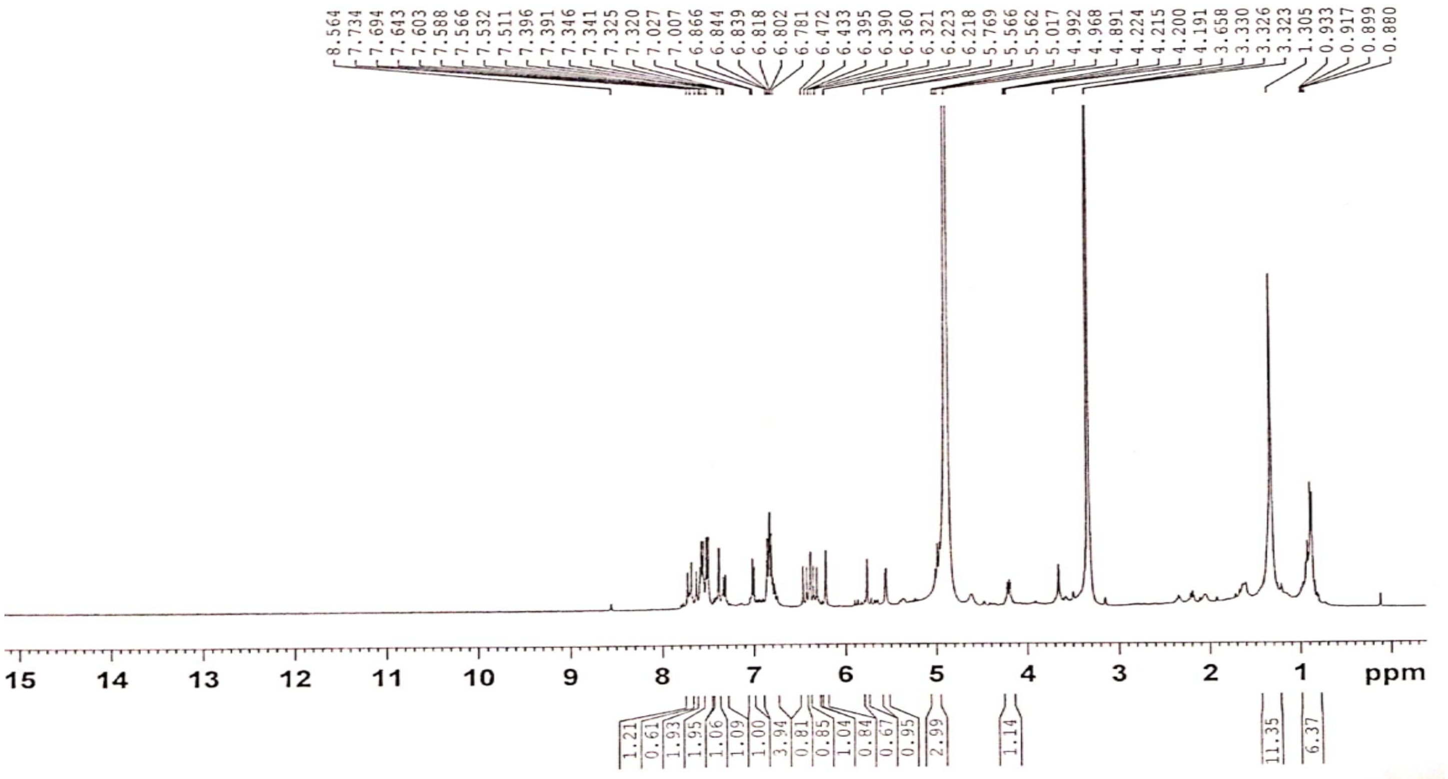

Figure S22. ^{1}H-NMR spectrum (400 MHz, CD_3OD) of quercetin 3-*O*-(2″,4″-di-*E*-p-coumaroyl)-α-L-rhamnopyranoside (compound **2**).

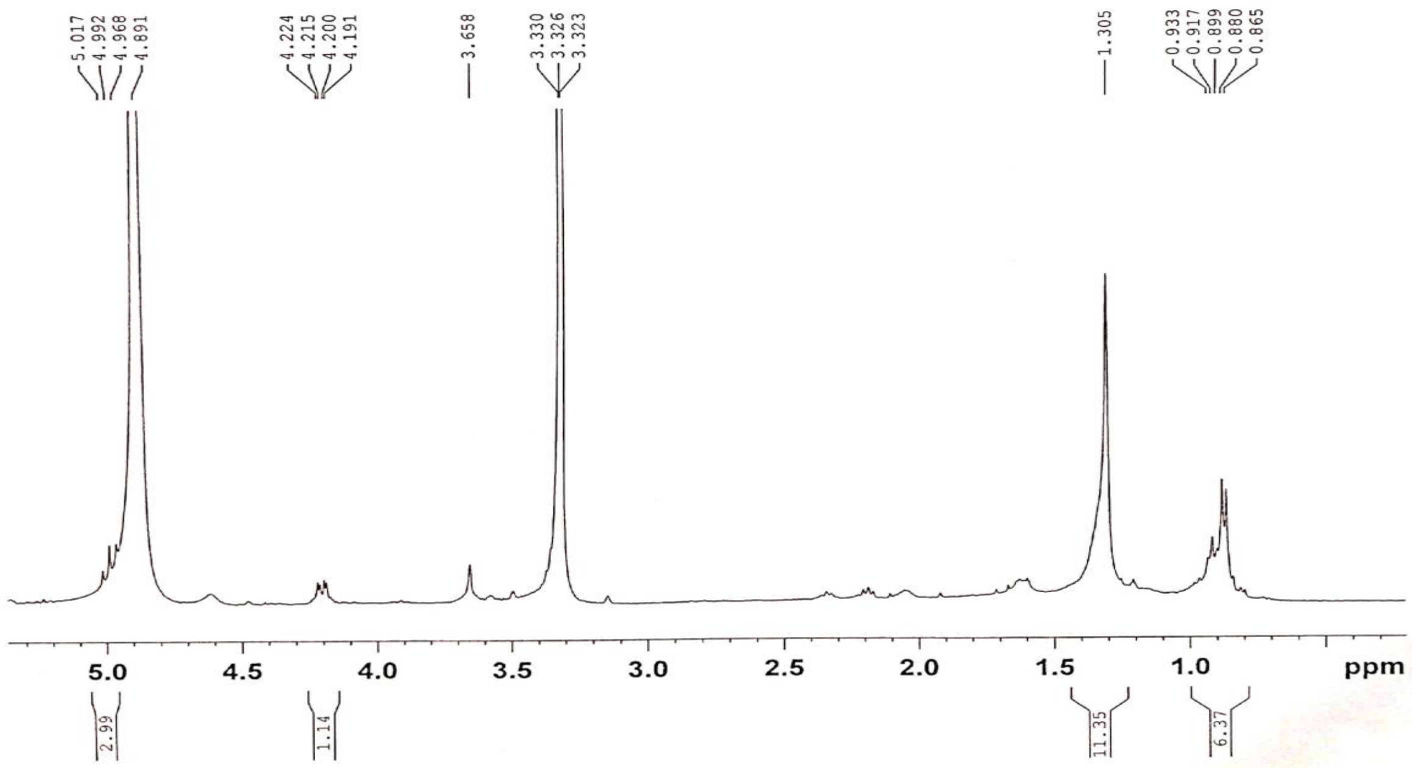

Figure S23. ^{1}H-NMR spectrum (400 MHz, CD_3OD) of quercetin 3-*O*-(2″,4″-di-*E*-p-coumaroyl)-α-L-rhamnopyranoside (compound **2**) (expanded).

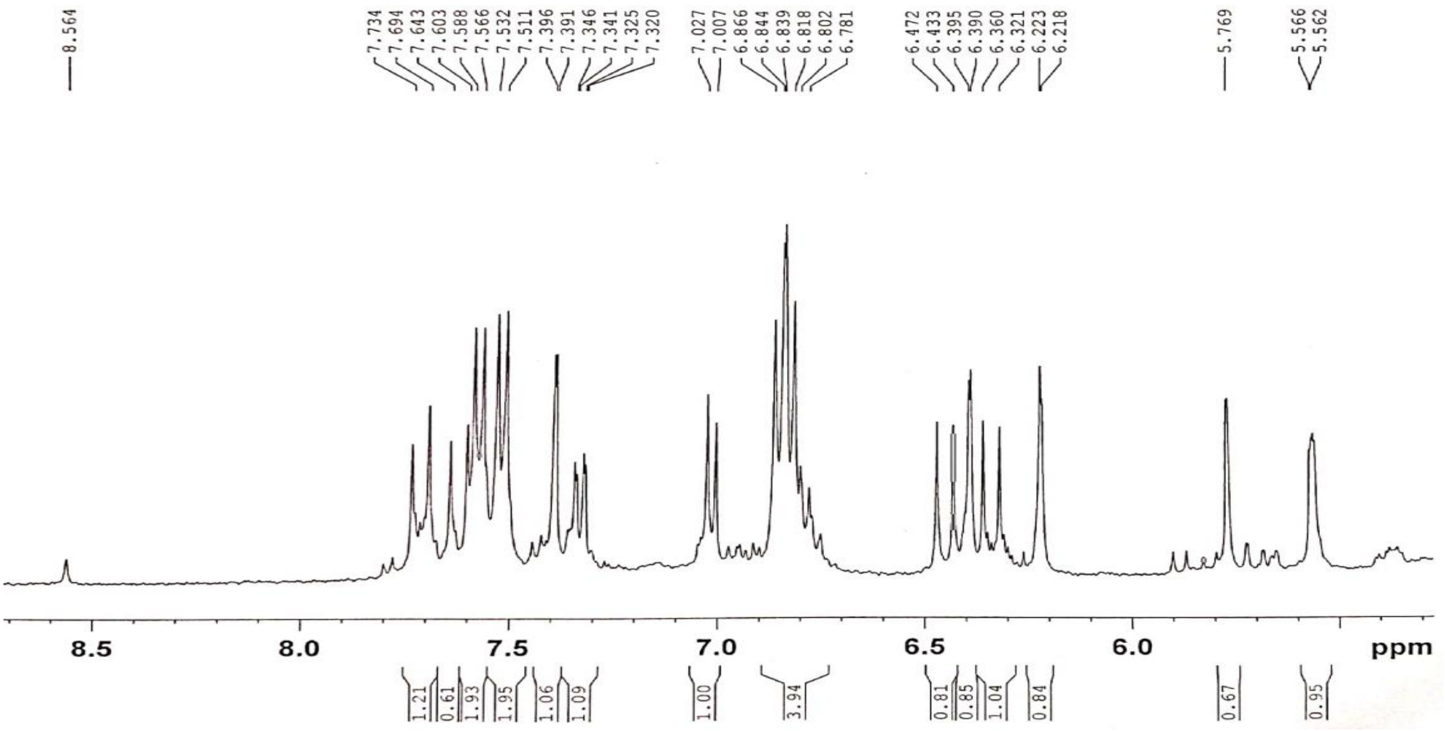

Figure S24. ^{1}H-NMR spectrum (400 MHz, CD_3OD) of quercetin 3-*O*-(2″,4″-di-*E*-p-coumaroyl)-α-L-rhamnopyranoside (compound **2**) (expanded).

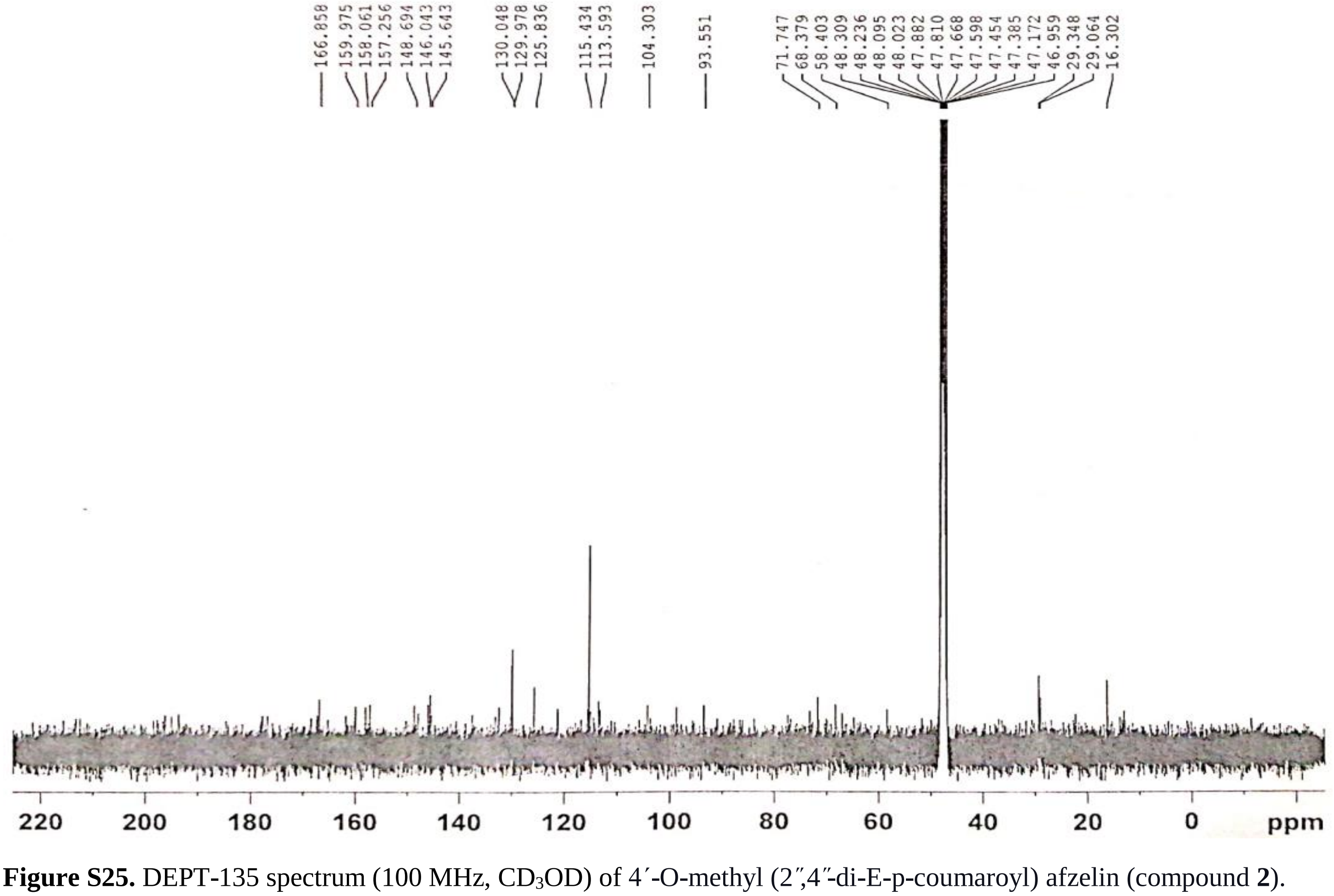

Figure S25. DEPT-135 spectrum (100 MHz, CD_3OD) of 4′-O-methyl (2″,4″-di-E-p-coumaroyl) afzelin (compound **2**).

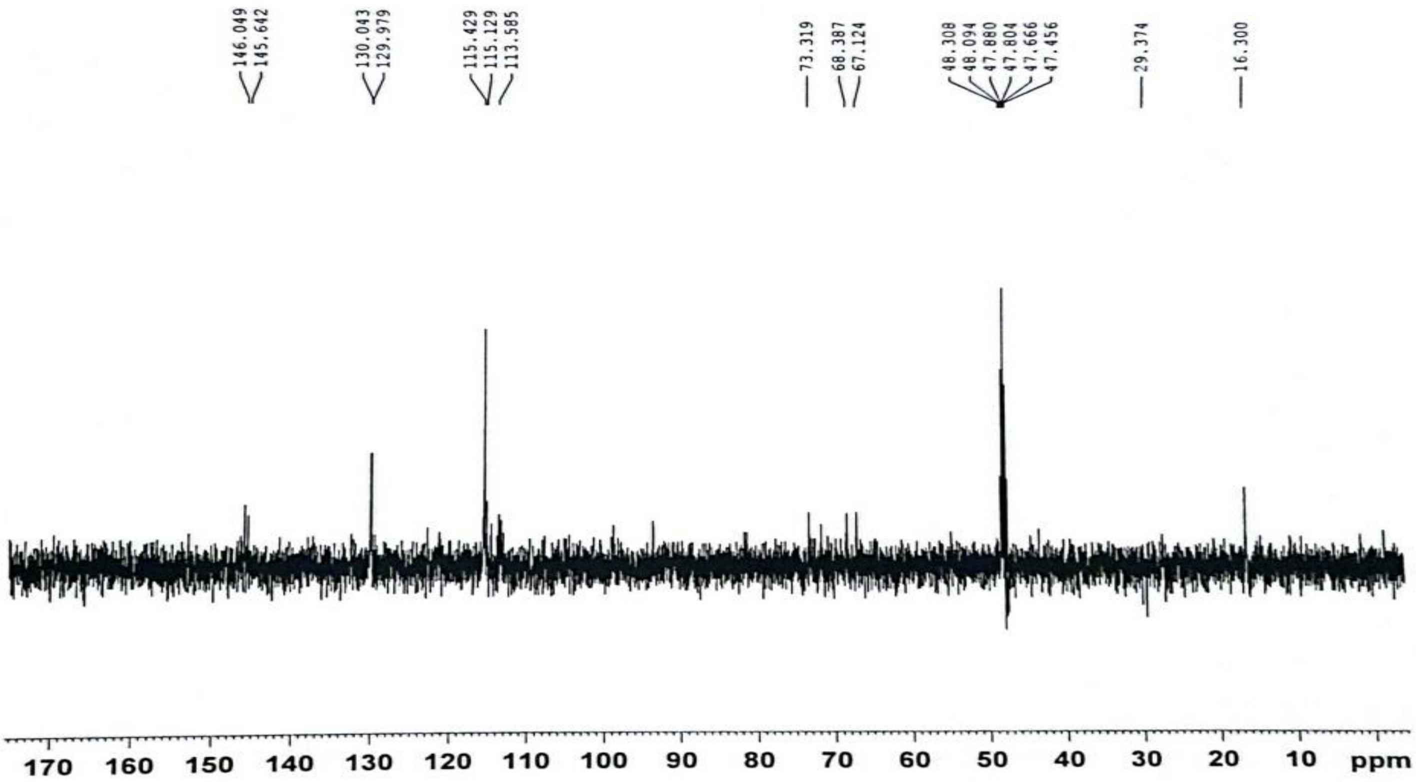

Figure S26. DEPT-135 spectrum (100 MHz, CD_3OD) of 4′-O-methyl (2″,4″-di-E-p-coumaroyl) afzelin (compound **2**).

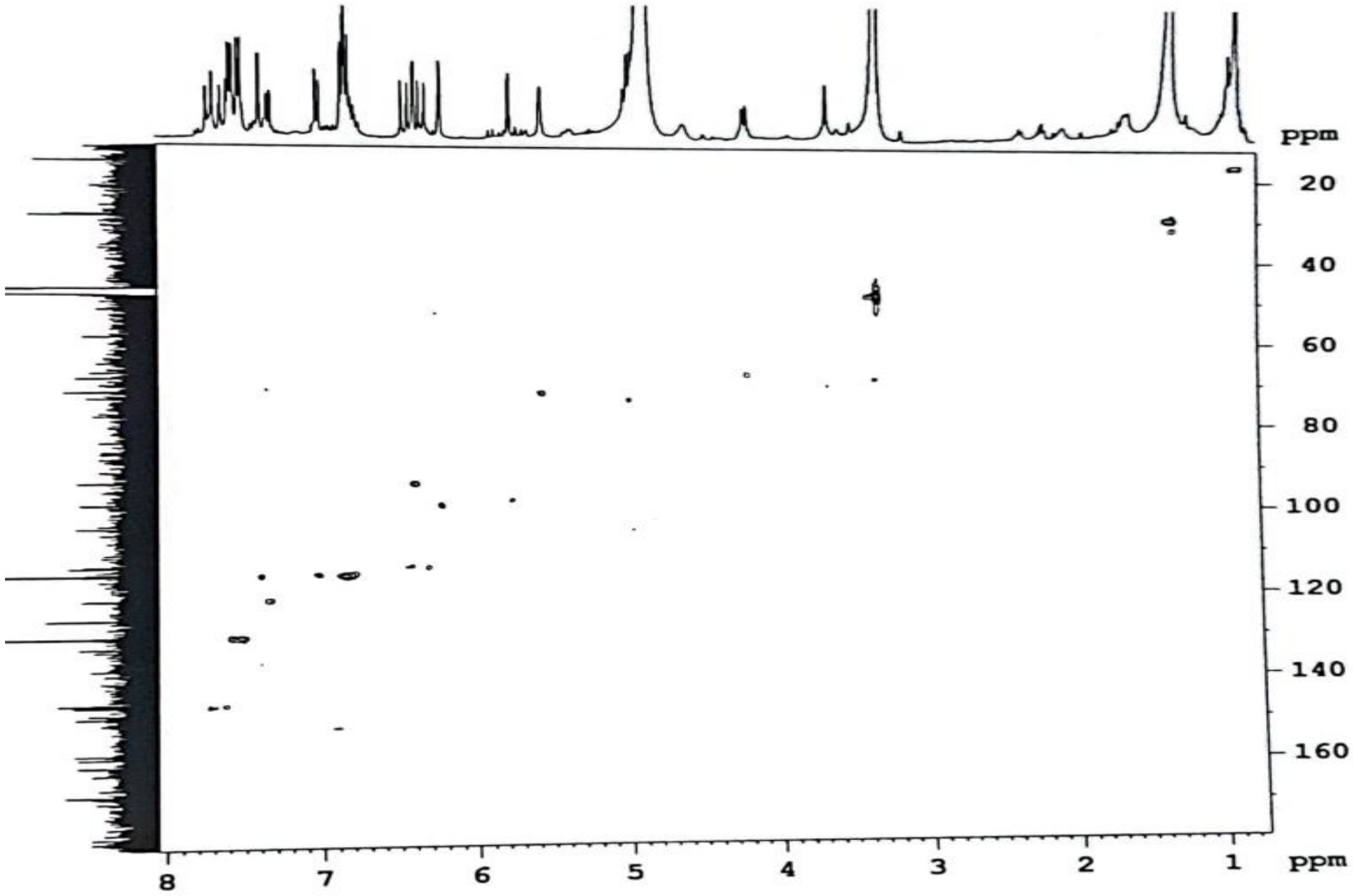

Figure S27. HSQC spectrum (400 MHz, CD_3OD) of 4′-O-methyl (2″,4″-di-E-p-coumaroyl) afzelin (compound **2**).

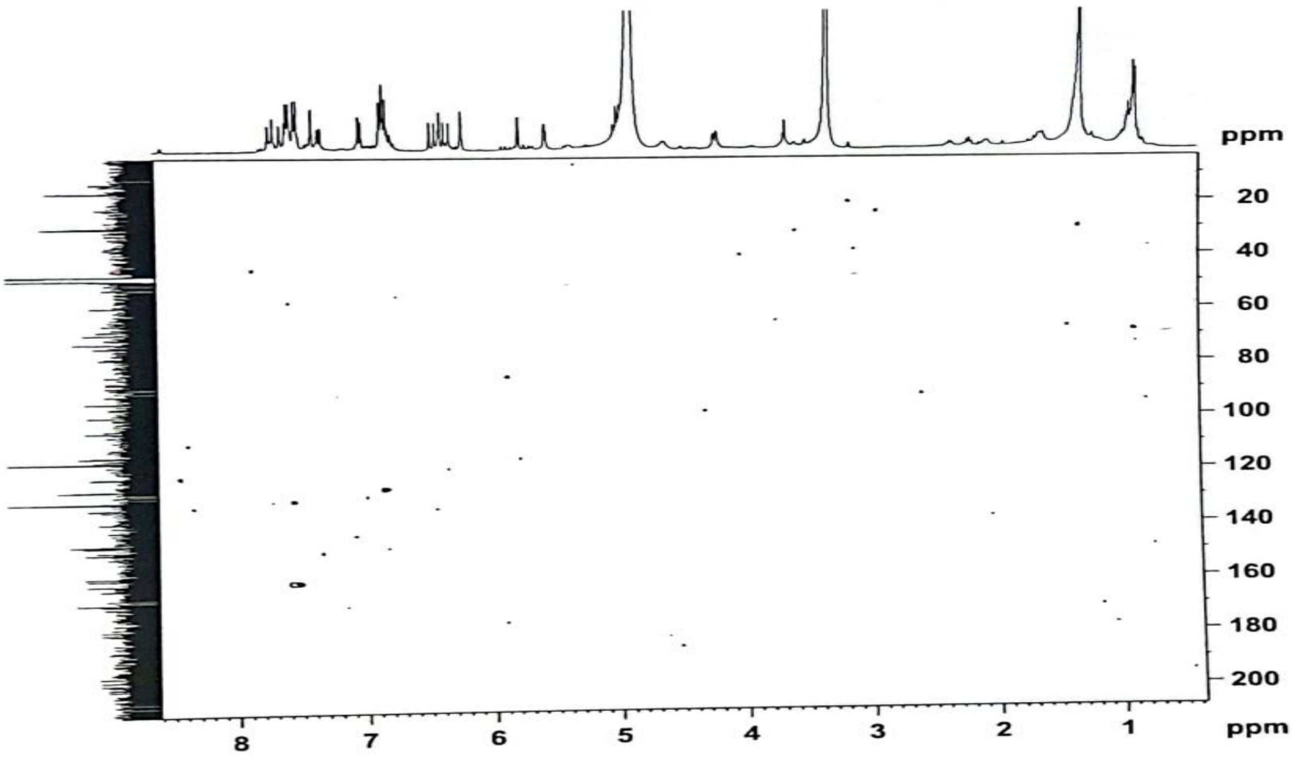

Figure S28. HMBC spectrum (400 MHz, CD_3OD) of 4′-O-methyl (2″,4″-di-E-p-coumaroyl) afzelin (compound **2**).

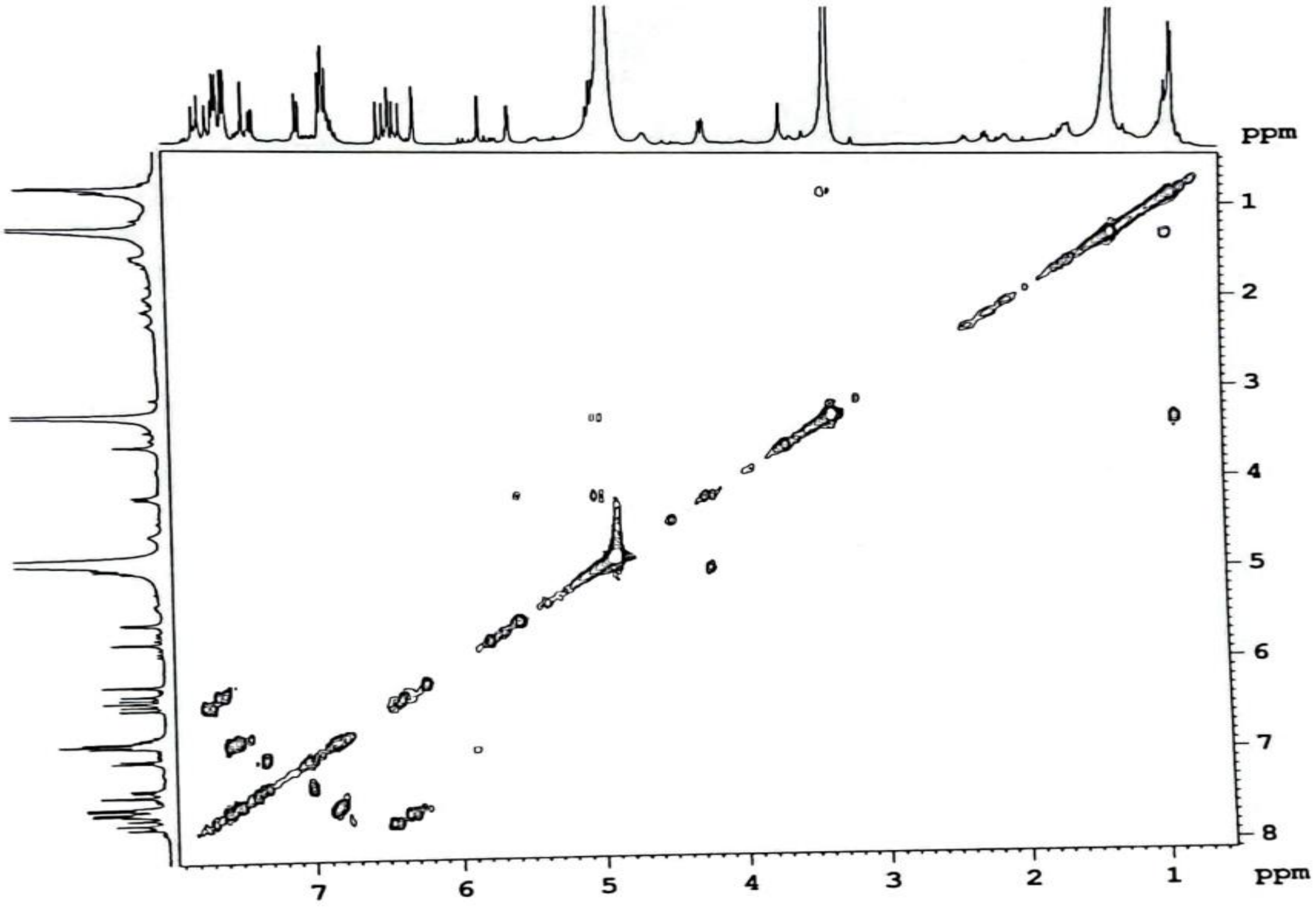

Figure S29. COSY spectrum (400 MHz, CD_3OD) of 4′-O-methyl (2″,4″-di-E-p-coumaroyl) afzelin (compound **2**).

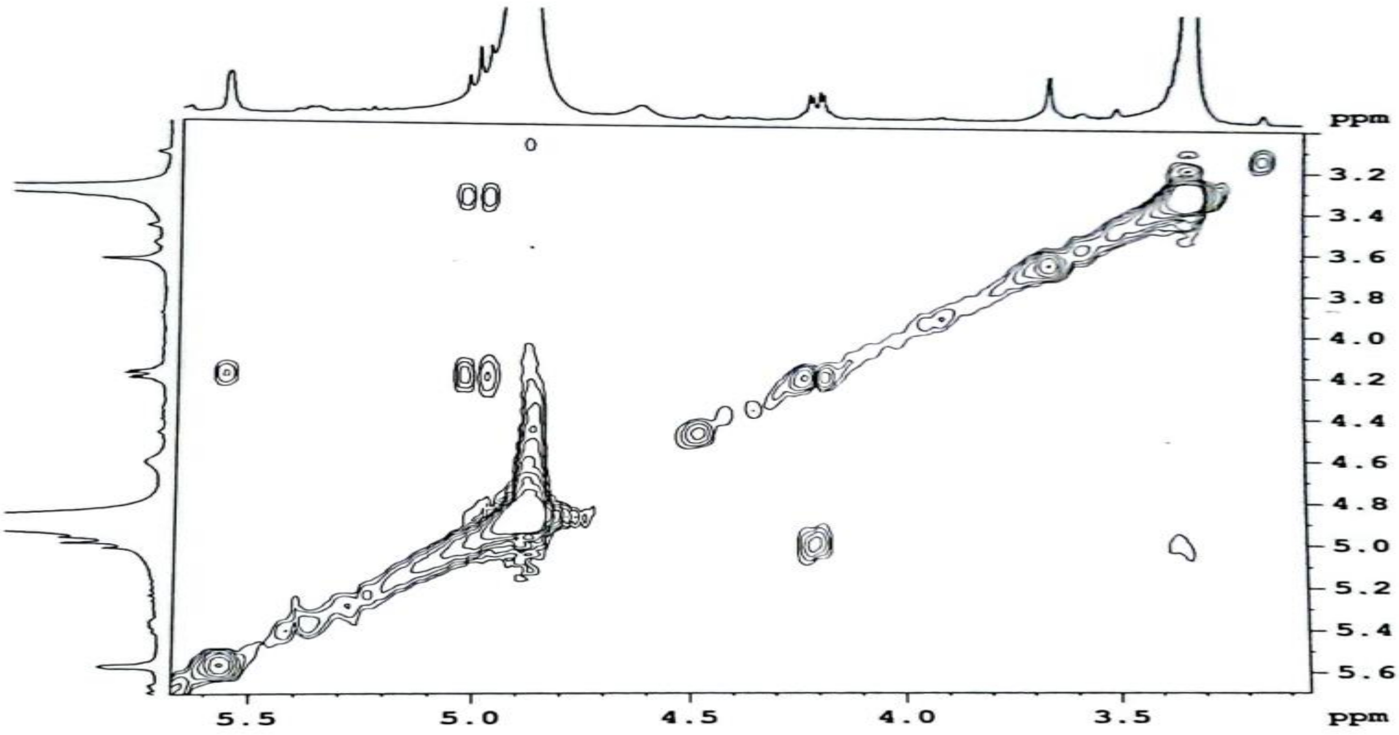

Figure S30. COSY spectrum (400 MHz, CD_3OD) of 4´-O-methyl (2″,4″-di-E-p-coumaroyl) afzelin (compound **2**) (expanded)

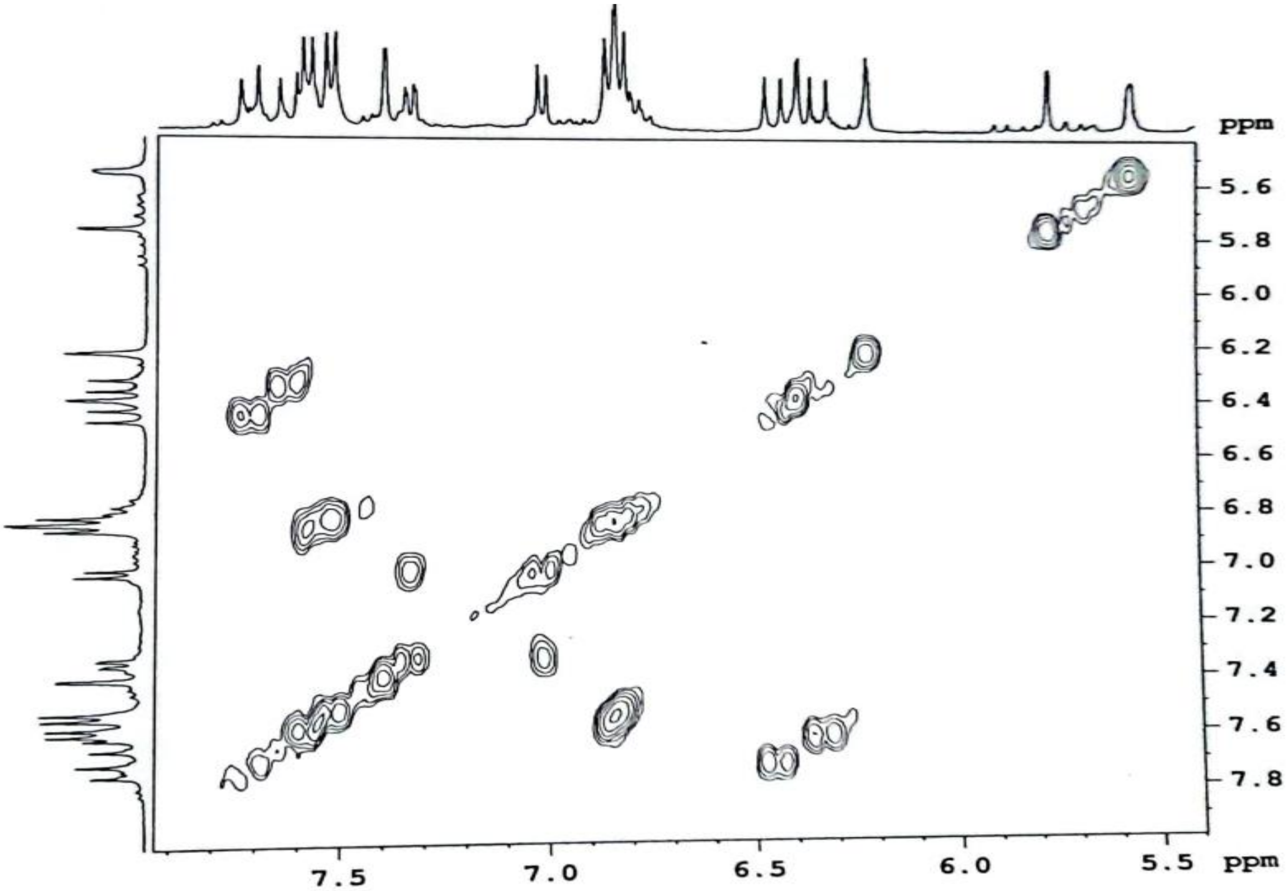

Figure S31. COSY spectrum (400 MHz, CD_3OD) of 4´-O-methyl (2″,4″-di-E-p-coumaroyl) afzelin (compound **2**) (expanded).

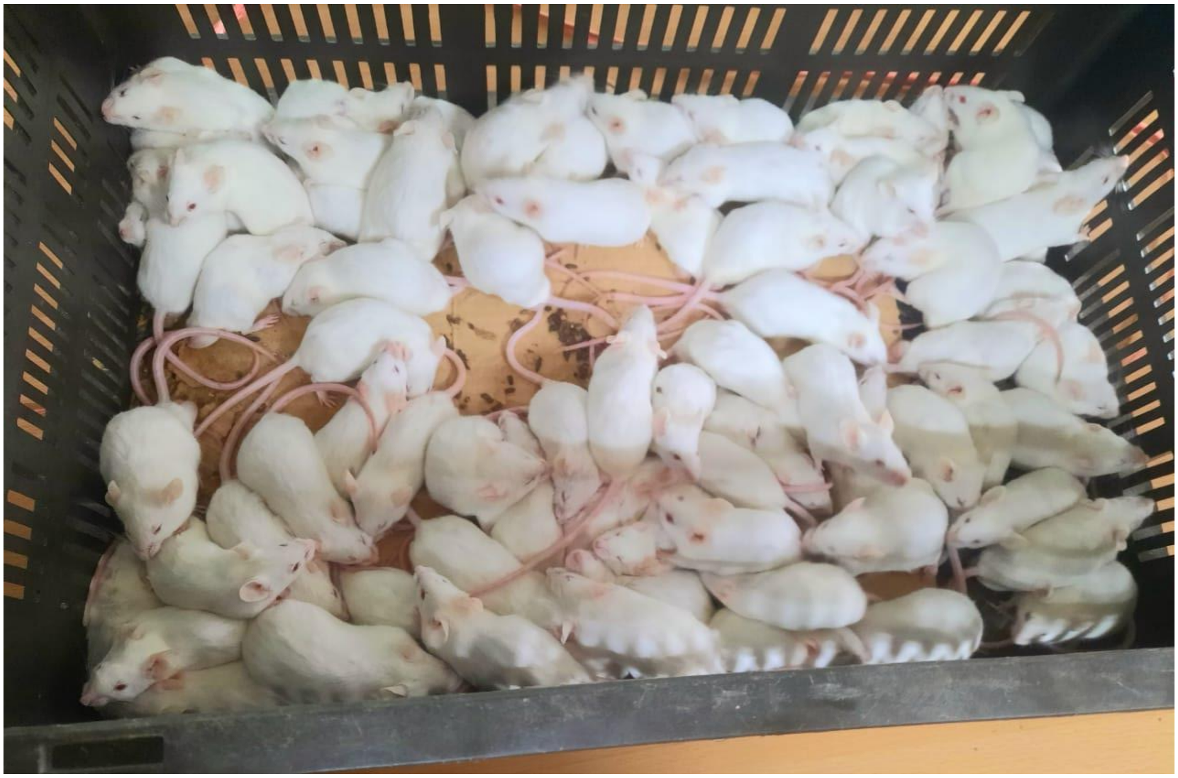

Figure S32. The collected Swiss Albino Mice for conducting pharmacological studies of *L. glutinosa*.
